# Artificial Intelligence in Periodontology: A Systematic Review

**DOI:** 10.1111/jre.70107

**Published:** 2026-03-30

**Authors:** Antonin Tichy, Nils Werner, Helena Dujic, Charlotte Wetzel, Vinay Pitchika, Caspar Victor Bumm, Matthias Folwaczny, Falk Schwendicke

**Affiliations:** ^1^ Department of Conservative Dentistry, Periodontology and Digital Dentistry LMU University Hospital, LMU Munich Munich Germany; ^2^ Institute of Dental Medicine First Faculty of Medicine of the Charles University Prague Czech Republic

**Keywords:** clinical decision support, deep learning, dental imaging, diagnostic accuracy, machine learning, periodontitis, risk prediction

## Abstract

**Aim:**

To provide a comprehensive review of artificial intelligence (AI) applications in periodontology, focusing (1) on deep learning for image‐based diagnosis of periodontitis and (2) on non‐image‐based AI applications across periodontal care.

**Methods:**

This study adhered to PRISMA guidance. Six databases (PubMed, Scopus, Web of Science, Embase/Ovid, IEEE Xplore, and arXiv) were searched. The first review question (PICO 1) focused on applications of deep learning to human imaging data for diagnosing periodontitis, and the systematic review was followed by a modified QUADAS‐2 risk‐of‐bias (RoB) assessment. The second part (PICO 2) scoped AI applications in periodontology using non‐imaging data. Because of substantial heterogeneity in tasks, inputs, and outcomes, PICO 2 was synthesized narratively without formal RoB assessment.

**Results:**

PICO 1 included 29 studies, predominantly using panoramic radiographs (*n* = 21). Binary periodontitis classification achieved accuracies of 81%–99% on panoramic radiographs and 78% on CBCT, whereas staging/severity showed lower performance (accuracy 64%–91% in panoramic radiographs; 83% in intraoral radiographs with AUROC 0.84–0.93). Photograph‐based screening achieved AUROC 0.93. RoB was generally low, but applicability concerns were frequent, mainly because of single‐center datasets. PICO 2 included 65 studies, covering diagnosis and classification of periodontitis (AUROC 0.77–0.85), risk stratification and screening (AUROC 0.60–0.98), progression, and treatment outcome modeling (AUROC 0.58–0.89), oral‐systemic associations, biomarker identification, and clinical data mining using natural language processing, which achieved near‐perfect metrics.

**Conclusion:**

Generalizability remains the key limitation across applications, driven by limited data diversity, inconsistent tasks/metrics, and scarce external testing. Future studies should prioritize multicenter evaluation, transparent reporting, and prospective assessments of workflow impact and patient‐related outcomes.

**Registration:** PROSPERO identification number CRD420251128758.

## Graphical abstract

AI shows promise across periodontology, with deep learning achieving strong performance for image‐based diagnosis of periodontitis. However, limited data diversity, inconsistent metrics, and scarce external validation raise concerns about generalizability and clinical applicability.

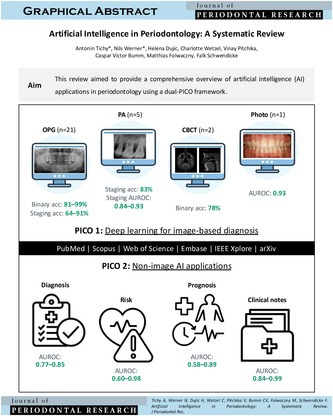

## 1 Introduction

Periodontitis is a chronic, non‐communicable disease primarily caused by the development of a dysbiotic microbial biofilm, which elicits a dysregulated immune response in a susceptible host. This further promotes an environmental niche for pathogenic bacteria, which in turn amplifies the inflammatory response, creating a vicious cycle resulting in irreversible tissue destruction [1, 2]. Periodontitis, which ultimately leads to tooth loss, affects nutritional intake, quality of life, and self‐esteem [3, 4]. Additionally, periodontitis has been associated with various systemic conditions, such as cardiovascular diseases or diabetes mellitus [3, 5, 6, 7, 8].

Diagnosis of periodontitis encompasses a clinical and radiographic examination, followed by initial cause‐related therapy (active periodontal care), including supra‐ and subgingival instrumentation and, if indicated, adjuvant antimicrobial medication and surgical procedures [9]. Treatment builds on routinely and accurately performed measurements to evaluate the acute periodontal status, including attachment loss, furcation involvement [10], tooth mobility [11], and bleeding on probing [12]. Clinical examination is commonly complemented by 2D radiography [13], whereas cone beam computed tomography (CBCT) [14, 15], ultrasound [16], magnetic resonance imaging [17], photographs, or intraoral scans [18] may further improve diagnosis and monitoring of periodontitis and individualization of therapy in specific cases. Albeit being central for prevention of further irreversible tissue damage, detecting the early phases of periodontitis or its recurrence remains challenging [19, 20].

The use of artificial intelligence (AI) offers promise for the detection of periodontitis, as machine learning methods are capable to learn complex patterns from data and support decision‐making [21, 22, 23, 24, 25, 26, 27, 28]. Shallow machine‐learning approaches (e.g., logistic regression, support vector machines, random forests, gradient boosting, artificial neural networks) leverage data obtained from clinical and/or radiographic examination, demographic features, risk factors and, where available, biomarkers to classify periodontitis status. They can also be used for predictive modeling which estimates individual risk trajectories and likelihood of periodontitis progression or recurrence [29]. Deep learning models [e.g., convolutional neural networks (CNNs), and transformers] are more complex, stacking many hidden layers. Although they require more computing power and are less interpretable, deep learning models are capable of recognizing specific features directly from high‐dimensional data (e.g., text, images), enabling not only classification, but also tasks like object detection and segmentation. Classification provides a single label per image (e.g., periodontitis present/absent; stage I–IV), object detection adds localization using a bounding box, and segmentation delineates the object on pixel level [30]. In practice, classification is easy to deploy but sacrifices spatial detail; object detection guides attention at modest additional cost; and segmentation provides detail but demands extensive annotation and higher computational power. Provided training on diverse, well‐annotated data, these AI methods can support diagnostic precision and personalized periodontal care [31].

Given the rapid advancement of AI technologies and the surge in their dental applications [32], this review aimed to provide a comprehensive overview of AI in periodontology. Several systematic reviews on this topic have already been published [21, 22, 23, 24, 25, 26, 27, 28]. Most of them focused on the diagnosis of gingivitis and periodontitis, with particular emphasis on periodontal bone loss in radiographs, but studies in photographs were also reviewed [21]. This review is divided into two parts. The focus of the first part is image‐based diagnostics of periodontitis using deep learning. Unlike previous reviews, we included not only 2D radiographs [23, 26, 27, 28], but also CBCTs, and in an attempt to reduce heterogeneity, which was previously reported to be very high, we excluded studies investigating only gingivitis and using only shallow machine learning. Additionally, strict eligibility criteria were applied to ensure the inclusion of high‐quality studies in terms of methodology and reporting. The second part explores other applications of AI in periodontology, which have received less attention, broadening the scope of this review beyond image analysis. By synthesizing the latest deep learning research, this review aims to highlight the strengths and limitations of AI models in periodontal diagnostics and guide future research to facilitate clinical translation.

## 2 Methods

A detailed protocol was develo ped in accordance with the PRISMA (Preferred Reporting Items for Systematic Reviews and Meta‐Analyses) 2020 statement [33]. The protocol was publicly registered in PROSPERO (CRD420251128758), and the systematic review was guided by the following PICOS features.

### 2.1 PICO 1: Deep Learning for Image‐Based Diagnosis of Periodontitis

#### 2.1.1 Eligibility Criteria

- Population (P): Humans undergoing periodontal assessment using various imaging modalities (e.g., intraoral radiographs, panoramic radiographs, CBCT, intraoral photographs, and ultrasound).
- Intervention (I): Deep‐learning models (e.g., CNNs, transformers) applied to image‐based periodontal diagnosis (e.g., bone loss, periodontal staging, and furcation defects).
- Comparator (C): Gold‐standard reference methods (e.g., data from clinical periodontal examination and expert consensus on the basis of radiographic assessment) for model evaluation.
- Outcomes (O): Diagnostic accuracy metrics such as sensitivity (recall), specificity, precision, accuracy, F1 score, or area under the receiver operating characteristic curve (AUROC). Given the heterogeneity of tasks and outcomes across included studies, we did not predefine a single primary metric applicable to all studies.
- Study design (S): Original diagnostic accuracy studies using human data (prospectively or retrospectively collected).

Studies in English were included. We excluded studies using only non‐imaging data (e.g., electronic health records, and biomarkers), studies performing only pre‐processing (e.g., landmark detection without a diagnostic task), studies focusing only on gingivitis or risk factors (e.g., biofilm and calculus detection) unless explicitly linked to the diagnostic process of periodontitis, studies assessing apical periodontitis and peri‐implantitis, studies using non‐human data, and in silico simulations. We also excluded case reports, reviews, editorials, letters (except for systematic reviews used for original study identification).

#### 2.1.2 Information Sources and Search

A scoping search was done on March 31, 2025; the records were independently screened by two reviewers (HD, AT), and the results informed the eligibility criteria. Then, a systematic search was conducted in six databases (PubMed, Scopus, Web of Science, Embase via Ovid, IEEE Xplore, and arXiv) with the last update on August 19, 2025. Search strings are summarized in Table 1. Additionally, the reference lists of identified systematic reviews and included articles were manually checked for additional studies.

**TABLE 1:** Search strings—PICO 1 (last update on August 19, 2025).

Database	Search string	Results (*n*)
PubMed	((“periodont*” OR “gingival margin” OR “alveolar bone loss”) AND (“intraoral radiograph*” OR “panoramic radiograph*” OR “CBCT” OR “cone beam” OR “ultrasound” OR “photograph*”) AND (“deep learning” OR “convolutional neural network*” OR “CNN” OR “transformer*” OR “artificial intelligence” OR “machine learning”) AND (“diagnostic accuracy” OR “sensitivity” OR “specificity” OR “precision” OR “recall” OR “F1 score” OR “AUC” OR “area under curve” OR “performance”))	153
Scopus	(TITLE‐ABS‐KEY (“periodont*” OR “gingival margin” OR “alveolar bone loss”) AND TITLE‐ABS‐KEY (“intraoral radiograph*” OR “panoramic radiograph*” OR “CBCT” OR “cone beam” OR “ultrasound” OR “photograph*”) AND TITLE‐ABS‐KEY (“deep learning” OR “convolutional neural network*” OR “CNN” OR “transformer*” OR “artificial intelligence” OR “machine learning”) AND TITLE‐ABS‐KEY (“diagnostic accuracy” OR “sensitivity” OR “specificity” OR “precision” OR “recall” OR “F1 score” OR “AUC” OR “area under curve” OR “performance”))	148
Web of Science	TS = ((“periodont*” OR “gingival margin” OR “alveolar bone loss”) AND (“intraoral radiograph*” OR “panoramic radiograph*” OR “CBCT” OR “cone beam” OR “ultrasound” OR “photograph*”) AND (“deep learning” OR “convolutional neural network*” OR “CNN” OR “transformer*” OR “artificial intelligence” OR “machine learning”) AND (“diagnostic accuracy” OR “sensitivity” OR “specificity” OR “precision” OR “recall” OR “F1 score” OR “AUC” OR “area under curve” OR “performance”))	116
Embase (via Ovid)	(periodontitis OR gingiva OR alveolar bone loss).ti,ab. (“intraoral radiograph*” OR “panoramic radiograph*” OR CBCT OR “cone beam” OR ultrasound OR photograph*).ti,ab. (“deep learning” OR “convolutional neural network” OR CNN OR transformer OR “artificial intelligence” OR “machine learning”).ti,ab. (“diagnostic accuracy” OR sensitivity OR specificity OR precision OR recall OR “F1 score” OR AUC OR “area under curve” OR performance).ti,ab. Combined: #1 AND #2 AND #3 AND #4	33
IEEE Xplore	(“deep learning” OR “convolutional neural network” OR CNN OR transformer) AND (“periodontal” OR “gingival margin” OR “alveolar bone loss”) AND (“radiograph” OR CBCT OR “cone beam” OR ultrasound OR photograph) AND (“diagnostic accuracy” OR sensitivity OR specificity OR “F1 score” OR AUC OR “area under the curve”)	12
arXiv	deep learning periodontal	9

#### 2.1.3 Study Selection and Data Extraction

All identified studies were uploaded to Rayyan [34], which was also used for deduplication. Then, two reviewers (AT, NW) independently screened titles and abstracts, as well as full texts. Cohen's kappa of 0.875 indicated very good agreement of the reviewers. Conflicts (*n* = 11) were resolved through discussion or a third reviewer (HD). Exclusion reasons were reported by both reviewers. Data extraction from the included studies was performed by two reviewers (NW, CW) in Rayyan, and conflicts were resolved by a third reviewer (AT). We extracted information about data (image type, dataset size and splits, origin, availability), objective (condition of interest and its classification), reference standard (number and experience of annotators), model development (task type and architecture), and outcomes (performance metrics and external testing).

#### 2.1.4 Risk of Bias and Applicability Concerns

Two reviewers (NW and CW) independently assessed the risk of bias and applicability concerns using the QUADAS‐2 (Quality Assessment of Diagnostic Accuracy Studies) tool. Signaling questions customized for this review and an additional domain were utilized for reporting bias (Table 2). Conflicts were resolved by a third reviewer (AT).

**TABLE 2:** QUADAS‐2 signaling questions.

Domain	Signaling questions
Patient selection	Risk of bias: Were clear inclusion/exclusion criteria applied? Was the dataset representative of the clinical spectrum of periodontal disease? Applicability concerns: Were the data representative of cases seen in clinical practice? Were they collected in multiple centers? Is the model expected to generalize well to other data?
Index test	Risk of bias: Were thresholds for classification pre‐specified and clearly explained? Was there any indication of data leakage between training and testing? Applicability concerns: Was the index test applied in a way that reflects intended use in practice (e.g., automated analysis without extensive pre‐processing)?
Reference standard	Risk of bias: Was the reference standard appropriate to verify the presence/absence of periodontitis (e.g., clinical periodontal examination and expert‐labeled radiographs)? Applicability concerns: Was the reference standard clinically relevant, that is, can it be considered as the gold standard in diagnosing periodontitis?
Flow and timing	Risk of bias: Was the data flow clearly described? Were all the included images accounted for in the final analysis?
Reporting bias and missing data	Were all relevant/pre‐specified diagnostic accuracy outcomes reported?

### 2.2 PICO 2: Non‐Image AI Applications in Periodontology

#### 2.2.1 Eligibility Criteria

- Population (P): Humans (adults and adolescents) diagnosed with periodontitis
- Intervention (I): AI systems for non‐image‐based applications in periodontal care. This could include clinical decision support on the basis of clinical parameters, risk prediction and disease progression modeling using clinical/anamnestic data, biomarker analysis from clinical samples, treatment planning and outcome prediction, monitoring in supportive periodontal therapy, patient stratification, automated periodontal charting, or data extraction from electronic health records.
- Comparator (C): Gold‐standard reference methods (e.g., data from clinical periodontal examination).
- Outcomes (O): Diagnostic accuracy metrics such as sensitivity (recall), specificity, precision, accuracy, F1 score, or AUROC. As in PICO 1, we did not predefine a single primary metric applicable to all studies.
- Study design (S): Original studies (observational, interventional, diagnostic, prognostic, and descriptive).

Studies in English were included. We excluded studies using only imaging data (as they were covered in PICO 1), studies on healthy individuals, studies focusing only on gingivitis and peri‐implantitis, studies assessing apical periodontitis, in vitro studies, and studies using non‐human data. Furthermore, we excluded bioinformatics studies using gene expression databases without clinical sample collection, educational applications of AI (e.g., using chatbots for answering exam questions), and studies focusing solely on statistical analysis without AI components. We also excluded abstracts without available full‐texts, case reports, editorials, letters, and reviews (except for systematic reviews used for original study identification).

#### 2.2.2 Information Sources and Search

A systematic search was conducted in six databases (PubMed, Scopus, Web of Science, Embase via Ovid, IEEE Xplore, and arXiv) on November 28, 2025. Search strings are summarized in Table 3. Additionally, the reference lists of identified systematic reviews and included articles were manually checked for additional studies.

**TABLE 3:** Search strings—PICO 2 (November 28, 2025).

Database	Search string	Results (*n*)
PubMed	((“periodont*”) AND (“artificial intelligence” OR “machine learning” OR “deep learning” OR “convolutional neural network*” OR “CNN” OR “transformer*” OR „natural language processing” OR “predictive modeling”) AND (“diagnostic accuracy” OR “sensitivity” OR “specificity” OR “precision” OR “recall” OR “F1 score” OR “AUC” OR “area under curve” OR “performance”))	555
Scopus	(TITLE‐ABS‐KEY (periodont*) AND TITLE‐ABS‐KEY (“artificial intelligence” OR “machine learning” OR “deep learning” OR “convolutional neural network” OR “CNN” OR “transformer” OR “natural language processing” OR “predictive modeling”) AND TITLE‐ABS‐KEY (“diagnostic accuracy” OR “sensitivity” OR “specificity” OR “precision” OR “recall” OR “F1 score” OR “AUC” OR “area under curve” OR “performance”))	566
Web of Science	(periodont* AND (artificial intelligence OR machine learning OR deep learning OR convolutional neural network OR CNN OR transformer OR natural language processing OR predictive modeling) AND (diagnostic accuracy OR sensitivity OR specificity OR precision OR recall OR F1 score OR AUC OR performance))	626
Embase (via Ovid)	#1 periodont*.ti,ab,kw. or exp. periodontitis/or exp. periodontal disease/	712
IEEE Xplore	#2 (“artificial intelligence” or “machine learning” or “deep learning” or “convolutional neural network*” or CNN or transformer* or “natural language processing” or “predictive modeling”).ti,ab,kw. or exp. artificial intelligence/or exp. machine learning/or exp. deep learning/	114
arXiv	#3 (“diagnostic accuracy” or sensitivity or specificity or precision or recall or “F1 score” or AUC or “area under curve” or performance).ti,ab,kw. or exp. diagnosis/or exp. diagnostic accuracy/	9

#### 2.2.3 Study Selection and Data Extraction

All identified studies were uploaded to Rayyan [34], which was also used for deduplication. Then, two reviewers (NW and CVB) independently screened titles and abstracts, as well as full texts. Cohen's kappa of 0.939 indicated almost perfect agreement of the reviewers. Conflicts (*n* = 14) were resolved through discussion or a third reviewer (CW). Exclusion reasons were reported by both reviewers. Data extraction from the included studies was performed by two reviewers (NW and CVB) in Rayyan, and conflicts were resolved by a third reviewer (CW). We extracted information about clinical task, sample size, data used, AI model, and performance metrics (including external testing, where applicable).

## 3 Results

### 3.1 PICO 1: Deep Learning for Image‐Based Diagnosis of Periodontitis

**FIGURE 1:**
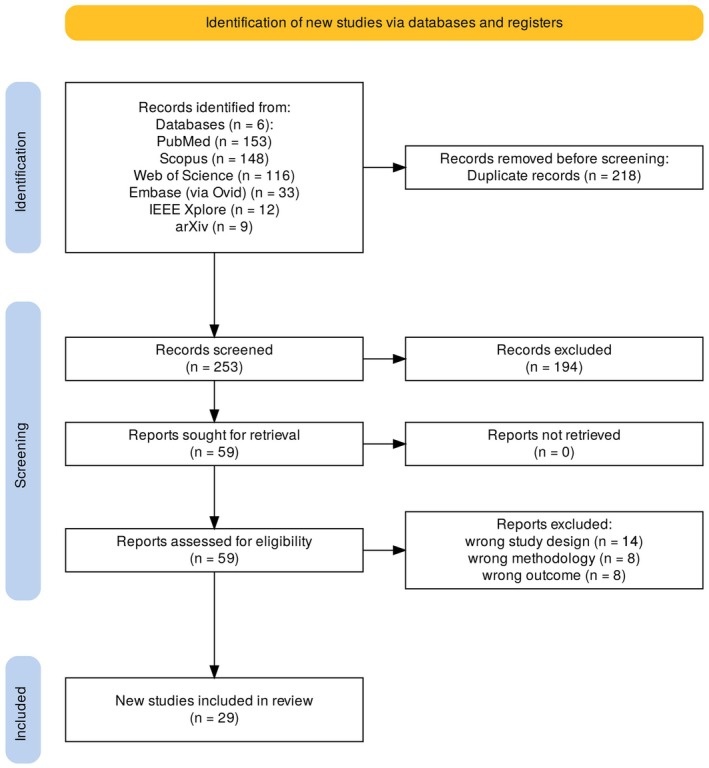
Prisma flow diagram—PICO 1.

The search in six databases identified 471 records. After 218 duplicates were removed, titles and abstracts of 253 studies were screened, resulting in the exclusion of 194 articles. Full‐text screening of 59 retrieved articles resulted in the exclusion of 30 studies, primarily because of incorrect study design (*n* = 14), methodology (*n* = 8), or outcomes (*n* = 8). Ultimately, 29 studies were included in the review. The PRISMA flow diagram is presented in Figure 1.

Most of the included studies used panoramic radiographs (*n* = 21) [13, 35, 36, 37, 38, 39, 40, 41, 42, 43, 44, 45, 46, 47, 48, 49, 50, 51, 52, 53, 54], followed by intraoral radiographs (*n* = 5) [55, 56, 57, 58, 59], CBCTs (*n* = 2) [60, 61], and intraoral scans/photographs (*n* = 1) [62]. More specifically, two studies used crops of panoramic radiographs [13, 42], panoramic views were generated from CBCT scans in another study [52], and Zhang et al. used CBCTs to determine the ground truth [53]. The dataset size ranged 85–12 179 panoramic radiographs, 700–21 819 intraoral radiographs, and 285–502 CBCTs (Table 4). The study by Tao et al. used screenshots of 387 intraoral scans for training and 183 intraoral photographs for external testing [62]. The datasets splits were mostly 70%–90% for training, with 10%–20% for validation and testing; only three studies used smaller splits for validation and/or testing [42, 47, 54]. Cross‐validation was used in six studies [13, 37, 40, 43, 53, 58]. The datasets originated from China (*n* = 8) [36, 38, 39, 45, 46, 52, 53, 62], Turkey (*n* = 7) [35, 41, 43, 44, 50, 58, 61], USA (*n* = 5) [42, 47, 55, 56, 61], South Korea (*n* = 2) [40, 54], UAE (*n* = 2) [51, 60], Germany (*n* = 2) [13, 57] and one each from Chile [42], Indonesia [49], Albania [48], India [37], and Thailand [59]. Three studies used publicly available data (Kaggle [50], Tufts [42], Dentex [47]) and only two of the 29 included studies [38, 51] made at least a part of their data available (Table 4).

**TABLE 4:** PICO 1—extracted information about data and annotations in the included studies (ordered by image type and year of publication).

Study	Image type	Dataset size	Data splits	Data origin	Data availability	Annotators
Kim et al., 2019 [54]	Panoramic radiographs	12 179	92–1.5‐6.5	South Korea	No	Five dental hygienists (5–19 years of experience) supervised by a board‐certified maxillofacial surgeon
Krois et al. [13]	Panoramic radiographs	85 (2001 cropped tooth segments)	70–30 (10‐fold cross‐validation)	Germany	No	Three dentists
Kurt‐Bayrakdar et al., 2020 [35]	Panoramic radiographs	2276 (1137 with alveolar bone loss; 1139 periodontally healthy)	80–10–10	Turkey	No	One oral and maxillofacial radiologist and one periodontologist (≥ 9 years of experience)
Li et al., 2021 [36]	Panoramic radiographs	506 from 2 institutions	80–20	China	No	Dentists (> 10 years of experience)
Jaiswal et al., 2022 [37]	Panoramic radiographs	500 (130 with periodontitis)	80–20 (3‐fold cross‐validation)	India	No	Multiple specialists (oral/maxillofacial radiologists, prosthodontists, surgeons, periodontists, and endodontists)
Kong et al., 2023 [38]	Panoramic radiographs	1747 (50 080 annotated teeth)	70–10–20	China	Yes	“Professional dentists”
Liu et al., 2023 [39]	Panoramic radiographs	Internal: 1924, External: 351	Internal: 66–20–14	China	No	Three periodontologists (> 5 years of experience) calibrated on 100 cases
Ryu et al., 2023 [40]	Panoramic radiographs	4083 (48 996 regions of interest)	80–20 (5‐fold cross‐validation)	South Korea	No	Two experts (trained for 2 months)
Uzun Saylan et al., 2023 [41]	Panoramic radiographs	685	80–10–10	Turkey	No	One oral and maxillofacial radiologist and one periodontologist (≥ 10 years of experience)
Cerda Mardini et al., 2024 [42]	Panoramic radiographs (crops)	500 (2010 crops of molars)	78–20–2	Chile, USA (Tufts)	No	One undergraduate student guided by a radiologist (> 5 years of experience)
Guler Ayyildiz et al., 2024 [43]	Panoramic radiographs	2533 (721 healthy, 842 stage 1/2, 970 stage 3/4)	80–20 (10‐fold random subsampling cross‐validation)	Turkey	No	Two calibrated investigators: (periodontologist with 12 years of experience and a specialty student)
Kurt‐Bayrakdar et al., 2024 [44]	Panoramic radiographs	1121 (30 369 labels—2251 total alveolar bone loss, 21 839 horizontal and 3464 vertical interdental bone loss, 2815 furcation defects)	80–10–10	Turkey	No	Three periodontologists and 1 oral and maxillofacial radiologist
Xue et al., 2024 [45]	Panoramic radiographs	320 Radiographs (8462 teeth)	90–10	China	No	Three senior dentists with background in periodontology and radiology (≥ 8 years of experience)
Yu et al., 2024 [46]	Panoramic radiographs	705	80–10–10	China	No	“Clinicians”
Feher et al., 2025 [47]	Panoramic radiographs	634	87–5–8	USA, Dentex	No	N/A (used clinical data)
Mathur et al., 2025 [48]	Panoramic radiographs	1874	Unclear (1874 for training, 200 for validation and testing)	Albania	No	One oral and maxillofacial radiologist and one periodontologist
Putra et al., 2025 [49]	Panoramic radiographs	500 (5390 teeth)	80–10–10	Indonesia	No	Radiologist and periodontologist
Resul et al., 2025 [50]	Panoramic radiographs	337	70–15–15 (5‐fold stratified shuffle splitting cross‐validation)	Turkey, Kaggle	No	One periodontologist
Rezallah et al., 2025 [51]	Panoramic radiographs	Internal: 817, External: 600	Internal: 80–20	UAE, Chile/USA	Partly (test dataset)	Oral and maxillofacial radiologists
Zeng et al. 2025 [52]	Panoramic radiographs (derived from CBCT)	2467	70–15–15	China	No	At least three dental professionals
Zhang et al., 2025 [53]	Panoramic radiographs (CBCT for reference standard)	506 (1568 teeth)	70–15–15 (6‐fold cross‐validation)	China	No	Two periodontologists (6 years of experience), a senior periodontologist consulted in case of disagreement
Kabir et al., 2021 [55]	Periapical radiographs	700	70–20–10	USA	No	Three examiners (two periodontologists, one resident in periodontology)
Lee et al., 2022 [56]	Periapical radiographs (full‐mouth series)	Internal: 693 (37 patients), External: 644 (46 patients)	Internal: 70–10–20	USA	No	Three calibrated examiners (two periodontologists, one resident in periodontology)
Hoss et al., 2023 [57]	Periapical radiographs	21 819	86–14	Germany	No	Seven calibrated dentists
Erturk et al., 2025 [58]	Bitewing radiographs	1752	80–20 (5‐fold cross‐validation)	Turkey	No	One observer (2 years of experience in oral radiology)
Piroonsan et al., 2025 [59]	Periapical and bitewing radiographs	1369 (791 periapicals, 578 vertical bitewings) from 556 patients	80–10–10	Thailand	No	N/A (used clinical data)
Shetty et al., 2024 [60]	CBCT (crops)	285 crops	80–20 (hold‐out test set of 85 crops)	UAE	No	One examiner (15 years of experience in dental radiology)
Kurt‐Bayrakdar et al., 2025 [61]	CBCT	502 volumes	80–10–10	Turkey, USA	No	Three experts in periodontology and oral and maxillofacial radiology (> 10 years of experience) set the reference standard, which was re‐checked by three experienced oral and maxillofacial radiologists.
Tao et al., 2026 [62]	Intraoral scans and intraoral photographs	Internal: 387 scans (13 images per scan), External: 183 photographs	Internal: 60–15–25	China	No	Internal: N/A (used clinical data); External: Two trained examiners, disagreements resolved by a third examiner.

*Note:* Links to publicly available datasets: Tufts ‐ http://tdd.ece.tufts.edu/, Dentex ‐ https://github.com/ibrahimethemhamamci/DENTEX, Kaggle—https://www.kaggle.com/datasets/kasmira/dentalpanoramic, Kong et al.—https://github.com/PuckBlink/PDCNN, Rezallah et al.—https://zenodo.org/records/4457648.

Although all articles investigated periodontitis, three of them [37, 52, 62] examined other dental conditions as well (Table 5). The predominant objective was bone loss assessment, which was used either as a basis for binary classification (presence/absence) of periodontitis (*n* = 11) [13, 35, 37, 39, 41, 48, 49, 50, 51, 54, 61], or to estimate bone loss severity and periodontitis staging (*n* = 13) [36, 38, 40, 42, 43, 45, 46, 51, 52, 55, 56, 57, 58]. The measurements were usually based on landmark detection. If presented, the coronal reference point for bone loss was either the cemento‐enamel junction (CEJ, *n* = 12) [13, 35, 36, 39, 40, 42, 46, 48, 52, 55, 56, 57] or 2 mm apical from CEJ (*n* = 5) [41, 44, 45, 49, 61]; the other reference points were the alveolar bone crest and root apex. Besides bone loss, studies investigated furcation involvement (*n* = 5) [38, 44, 53, 60, 61], periodontal stability on the basis of pocket depth [47], and three‐wall intrabony defects [59]. Annotations were generally performed by at least two experts or trained dental professionals, although some studies used a single annotator [42, 50, 58, 60]. Clinical data were used instead of annotations in three studies [47, 59, 62] (Table 4).

**TABLE 5:** PICO 1—extracted information about tasks, models, and performance metrics in the included studies (ordered by image type and year of publication).

Study	Condition of interest	Classification	Coronal reference point	Task type	Model architecture	Metrics
Kim et al., 2019 [54]	Periodontitis	Binary (healthy vs. bone loss)	Unclear	Segmentation, classification	CNN (DeNTNet ‐ U‐shape architecture + classifier)	Sensitivity 0.77, specificity 0.95, precision 0.73, AUROC 0.95, F1‐score 0.75
Krois et al. [13]	Periodontitis	Binary (healthy vs. bone loss defined as ≥ 20%)	CEJ	Classification	CNN	Sensitivity 0.81 ± 0.04, specificity 0.81 ± 0.05, accuracy 0.81 ± 0.02.
Kurt‐Bayrakdar et al., 2020 [35]	Periodontitis	Binary (healthy vs. bone loss)	CEJ	Classification	GoogleNet Inception v3 CNN	Sensitivity (recall) 0.94, specificity 0.89, precision 0.89, accuracy 0.91, F1‐score 0.92
Li et al., 2021 [36]	Periodontitis	Staging (healthy, mild, moderate, severe)	CEJ	Segmentation	R‐CNN + XGBoost	Suzhou dataset: Macro accuracy: 0.90, F1‐score 0.89. Zhongshan dataset: Macro accuracy 0.82, F1‐score 0.82.
Jaiswal et al., 2022 [37]	Caries, periodontitis, apical periodontitis, tooth wear, missing teeth, and impacted teeth	Binary (yes/no)	Unclear	Classification	Mask RCNN and ResNet101 (for periodontal bone loss)	Sensitivity (recall) 0.93, specificity 0.93, precision 0.82, accuracy 0.93, F1‐score 0.87
Kong et al., 2023 [38]	Periodontitis	Bone loss: staging (healthy, mild, moderate, severe); Furcation involvement: staging (healthy, mild, severe)	Unclear	Classification	Two‐stage convolutional detector (PDCNN) compared with RetinaNet, YOLOv4, CenterNet, and Faster R‐CNN	Mean average precision 0.78, accuracy 0.76
Liu et al., 2023 [39]	Periodontitis	Binary (healthy vs. periodontitis)	CEJ	Classification	PAR‐CNN (on the basis of AlexNet)	Internal: sensitivity (recall) 0.80, specificity 0.78, accuracy 0.79, AUROC 0.84; External: sensitivity (recall) 0.82, specificity 0.78, accuracy 0.80, AUROC 0.79
Ryu et al., 2023 [40]	Periodontitis	Bone loss severity (normal, moderate, severe, edentulous)	CEJ	Object detection	Faster R‐CNN	Sensitivity (recall) 0.90, precision 0.90, F1‐score 0.90, AUROC 0.91
Uzun Saylan et al., 2023 [41]	Periodontitis	Binary (yes/no)	CEJ‐2 mm	Segmentation	YOLOv5	Sensitivity (recall) 0.75, precision 0.76, F1‐score 0.76
Cerda Mardini et al., 2024 [42]	Periodontitis	Bone loss staging (< 15%, 15%–33%, > 33%)	CEJ	Landmark detection	DCNN (Xception‐based)	Macro averaged sensitivity (recall) 0.30, specificity 0.65, precision 0.14, accuracy 0.64, F1‐score 0.20
Guler Ayyildiz et al., 2024 [43]	Periodontitis	Bone loss staging (healthy, stage I‐II, stage III‐IV)	Unclear	Classification	CNNs (ResNet50, DenseNet121, InceptionV3) with/without additional features and machine‐learning classifiers	Best‐performing model: sensitivity (recall) 0.83, specificity 0.94, precision 0.88, accuracy 0.91, F1‐score 0.86, AUROC 0.89
Kurt‐Bayrakdar et al., 2024 [44]	Periodontitis	Bone loss patterns (total, horizontal, vertical); Furcation defects	CEJ‐2 mm	Segmentation	CNN (U‐Net)	Total alveolar bone loss: sensitivity (recall) 1.00, precision 1.00, accuracy 0.99, F1‐score 1.00, AUROC 0.95. Horizontal bone loss: sensitivity (recall) 0.95, precision 0.94, accuracy 0.89, F1‐score 0.94, AUROC 0.91. Vertical bone loss: sensitivity (recall) 0.56, precision 0.85, accuracy 0.51, F1‐score 0.67, AUROC 0.73. Furcation defects: sensitivity (recall) 0.89, precision 0.93, accuracy 0.84, F1‐score 0.91, AUROC 0.87.
Xue et al., 2024 [45]	Periodontitis	Bone loss staging (< 15%, 15%–33%, > 33%)	CEJ‐2 mm	Landmark detection	Ensemble of YOLOv8, Mask R‐CNN, and TransUNet	Accuracy 0.90
Yu et al., 2024 [46]	Periodontitis	Bone loss staging (< 15%, 15%–33%, > 33%)	CEJ	Segmentation	SegFormer (MiT‐B3 transformer backbone)	Stage I: sensitivity (recall) 0.62–0.65, specificity 0.95, precision 0.77, F1‐score 0.68–0.70; Stage II: sensitivity (recall) 0.79–0.80, specificity 0.73, precision 0.73–0.74, F1‐score 0.76; Stage III: sensitivity (recall) 0.77–0.80, specificity 0.90–0.91, precision 0.78–0.81, F1‐score 0.79
Feher et al., 2025 [47]	Periodontitis	Periodontal stability (healthy ‐ PPD < 3 mm, stable ‐ < PPD, unstable ‐ PPD ≥ 5 mm)	N/A	Classification	ResNet 50	Healthy: F1‐score 0.45, AUROC 0.71; stable: F1‐score 0.57, AUROC 0.56; unstable: F1‐score 0.54, AUROC 0.67.
Mathur et al., 2025 [48]	Periodontitis	Binary (healthy vs. bone loss)	CEJ	Classification	VGG‐16 CNN	Sensitivity (recall) 0.83, specificity 0.87, precision 0.89, accuracy 0.89, F1‐score 0.86
Putra et al., 2025 [49]	Periodontitis	Binary (healthy vs. bone loss)	CEJ‐2 mm	Object detection	YOLOv8	Sensitivity (recall) 0.95, specificity 0.81, precision 0.91, accuracy 0.90, F1‐score 0.93
Resul et al., 2025 [50]	Periodontitis	Binary (yes/no)	N/A	Classification	APD‐FFNet (feature fusion model) compared with DenseNet201, ResNet152V2, VGG16, InceptionResNetV2, InceptionV3, MobileNet, and Vision transformer models	Sensitivity (recall) 0.93, precision 0.95, accuracy 0.94, F1‐score 0.94, AUROC 0.93, Jaccard similarity coefficient 0.88, Matthews correlation coefficient 0.88
Rezallah et al., 2025 [51]	Periodontitis	Binary (healthy vs. bone loss), Bone loss severity (mild, moderate, severe).	N/A	Object detection	MobileNetV2 + YOLOv8	Binary (MobileNetV2): sensitivity (recall) 1.00, accuracy 0.88; Severity (YOLOv8): sensitivity (recall) 0.70, precision: 0.74
Zeng et al. 2025 [52]	Dental caries, wisdom teeth, periradicular lesions, periodontitis	Staging (normal, mild, moderate, severe)	CEJ	Segmentation	MMC‐YOLO with MPFAM, CGFM, ESFM, IPS module + MDD	Periodontitis staging accuracy: normal 0.87, mild 0.91, moderate 0.77, severe 0.85
Zhang et al., 2025 [53]	Periodontitis	Furcation involvement (Glickman classification)	N/A	Classification	MLP, CNN (VGGNet and GoogleNet), Vision transformer	Best‐performing model (ViT): sensitivity (recall) 0.92, precision 0.98, accuracy: 0.92, F1‐score 0.95, AUROC 0.98
Kabir et al., 2021 [55]	Periodontitis	Bone loss staging (< 15%, 15%–33%, > 33%)	CEJ	Segmentation and classification	HYNETS (on the basis of U‐Net)	Periodontitis stage assignment: AUROC 0.97
Lee et al., 2022 [56]	Periodontitis	Bone loss staging (< 15%, 15%–33%, > 33%)	CEJ	Segmentation and classification	U‐Net‐based models	No bone loss: sensitivity (recall) 0.96, specificity 1.00, accuracy 0.99, AUROC 0.98; Stage I: sensitivity (recall) 0.82, specificity 0.97, accuracy 0.91, AUROC 0.89; Stage II: sensitivity (recall) 0.93, specificity 0.86, accuracy 0.88, AUROC 0.90; Stage III: sensitivity 0.80, specificity 0.99, accuracy 0.99, AUROC 0.90. External data: accuracy ‐ case diagnosis 0.85; stage = 0.87.
Hoss et al., 2023 [57]	Periodontitis	Bone loss staging (< 15%, 15%–33%, > 33%)	CEJ	Classification	CNNs (ResNet‐18, MobileNetV2, ConvNeX – small, base, large)	Overall sensitivity (recall) 0.89–0.91, specificity 0.66–0.71, accuracy 0.82–0.85, AUROC 0.88–0.91
Erturk et al., 2025 [58]	Periodontitis	Bone loss staging (< 15%, 15%–33%, > 33%)	Unclear	Classification	YOLOv8	Sensitivity (recall) 0.81, precision 0.82, accuracy 0.83, F1‐score 0.81
Piroonsan et al., 2025 [59]	Periodontitis	Intrabony defects: binary (three‐wall vs. non‐three‐wall defects)	N/A	Classification	InceptionV3, Inception ResNetV2, ResNet50V2, MobileNetV3Large, EfficientNetV2B1, VGG19	Best‐performing model (VGG‐19): sensitivity (recall) 0.82, specificity 0.67, precision 0.78, negative predictive value 0.88, accuracy 0.75, F1‐score 0.75
Shetty et al., 2024 [60]	Periodontitis	Furcation involvement: binary (yes/no)	N/A	Classification	ResNet101V2	Test accuracy 0.92
Kurt‐Bayrakdar et al., 2025 [61]	Periodontitis	Bone loss (various types of defects), Furcation defects	CEJ‐2 mm	Segmentation and classification	nnU‐Net v2, 3D U‐Net	Total bone loss: AUROC 0.85, furcation defects: AUROC 0.68 (0.81 in crops); accuracy across categories 0.99. Binary classification: sensitivity (recall) 0.76, specificity 0.79, precision 0.80, accuracy 0.78, F1‐score 0.78
Tao et al., 2026 [62]	Gingivitis, periodontitis	Healthy vs. gingivitis or stage I vs. stage II‐IV periodontitis	N/A	Classification	ResNet50‐based	Internal: AUROC 0.93; External: sensitivity (recall) 0.85, specificity 0.92, AUROC 0.93

Abbreviations: AUROC, area under the receiver operating characteristic curve; CEJ, cemento‐enamel junction; CNN, convolutional neural network; N/A, not applicable; ViT, vision transformer.

Although classification was most common, some studies employed segmentation (*n* = 8) [36, 41, 44, 46, 52, 54, 55, 56, 61] and object detection (*n* = 4) [40, 49, 51, 59] as well. Model architectures varied widely; some studies combined CNNs with shallow machine‐learning methods for the final classification step (Table 5). The reported metrics were inconsistent, making data synthesis complicated. In binary classification of periodontitis, the accuracy ranged from 81% [13] to 99% [44] on panoramic radiographs and was 78% on CBCT scans [61]. In staging, accuracy was lower compared to binary classification on both panoramic radiographs (64% [42]–91% [43]) and intraoral radiographs (accuracy 83% [58]; AUROC 84% [57]–93% [55]). On intraoral photographs, Tao et al. reported an AUROC of 93% for distinguishing healthy gingiva, gingivitis, and periodontitis [62]. Furcation defects were detected with an accuracy of 84% on panoramic radiographs [44], 68% in whole CBCTs [61], and 81% [61] to 91% [60] in cropped CBCTs. External testing on data from another source was performed only in four studies [39, 51, 56, 62].

**FIGURE 2:**
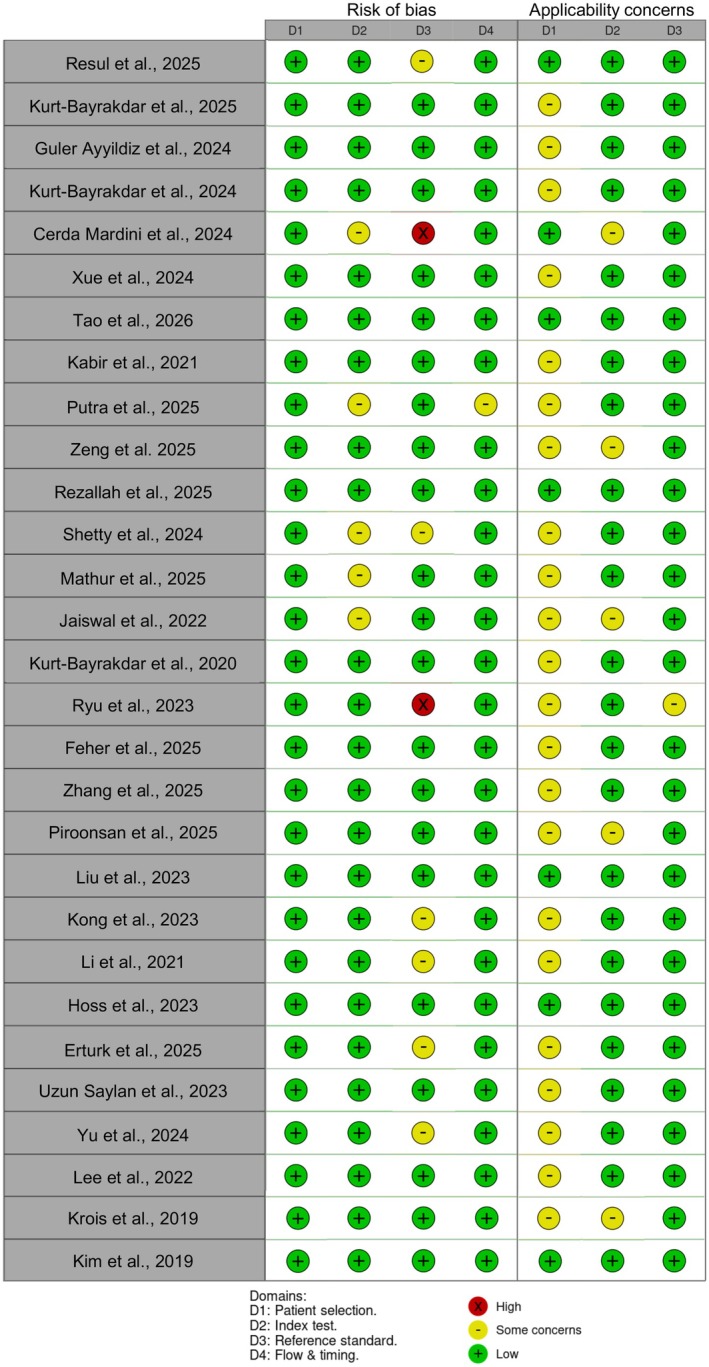
QUADAS‐2 summary plot.

Overall, the risk of bias was low, as indicated by the QUADAS‐2 summary plot (Figure 2). In terms of the index test, five studies used a small test set and/or had a risk of training data leakage into the test set [37, 42, 48, 49, 60]. The reference standard description was judged as insufficient in two studies [40, 42] and some concerns were found in another six [36, 38, 46, 50, 58, 60] mostly because they involved a single annotator [42, 50, 58, 60]. Flow and timing were well described, except for some unclarities in one study [49]. More concerns were identified in applicability, especially in patient selection, as most studies used data from a single center. Concerns related to the index test were identified in two studies which did not reflect intended use in practice (significant pre‐processing of the images was required for the model) [13, 42], the model performed well only on selected subsets [53] and in two studies, which focused on multiple tasks [37, 52]. Finally, the reference standard applicability was a concern in one study, which used a classification that is not used clinically [40]. Reporting bias was judged to be low in all studies.

### 3.2 PICO 2: Non‐Image AI Applications in Periodontology

**FIGURE 3:**
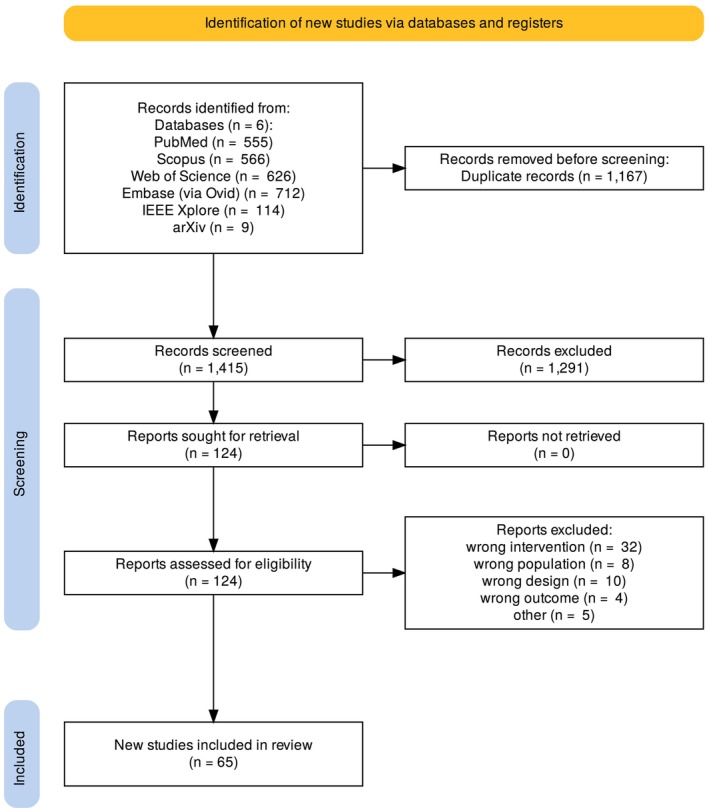
Prisma flow diagram—PICO 2.

The search identified 2582 records. After 1167 duplicates were removed, titles and abstracts of 1415 studies were screened, resulting in the exclusion of 1291 articles. Full‐text screening of 124 retrieved articles resulted in the exclusion of 59 studies because of incorrect intervention (*n* = 32), study design (*n* = 10), population (*n* = 8), outcome (*n* = 4), and other (*n* = 5). Ultimately, 65 studies were included in the review. The PRISMA flow diagram is presented in Figure 3. Extracted data for all following sections are presented in Table S1.

#### 3.2.1 Diagnosis and Classification (Using Non‐Imaging Inputs)

Twelve studies [63, 64, 65, 66, 67, 68, 69, 70, 71, 72, 73] applied various shallow machine learning models to classify periodontitis using non‐imaging inputs, usually structured clinical parameters (e.g., pocket depth, clinical attachment loss, mobility, and periodontal indices), demographics, and risk factors. The included studies often reported (almost) perfect performance with internal accuracy up to 100% in several single‐cohort studies. However, this could be caused by model overfitting, as relatively small datasets (≤ 1000) were used in 9 of the studies [63, 64, 65, 66, 68, 69, 70, 71, 72, 74]. External testing resulted in a marked performance decrease [66, 67], and even recent studies with larger sample size reported lower accuracy in binary periodontitis classification (AUROC 0.77–0.85) [73, 74]. One study examined the classification of periodontitis on the basis of natural language processing (NLP) and achieved overall accuracies of 66% and 72% in staging and grading on a test set of 32 unseen patients [71]. However, model performance was strongly imbalanced across individual stages and grades (Table S1).

#### 3.2.2 Risk Stratification, Screening and Clinical Decision Support

Twenty studies [75, 76, 77, 78, 79, 80, 81, 82, 83, 84, 85, 86, 87, 88, 89, 90, 91, 92, 93, 94] targeted screening, risk prediction, and patient stratification, spanning models combining periodontal measures with systemic parameters, laboratory markers, sociodemographic variables, and data from national health surveys or questionnaires, such as the CDC/AAP (Centers for Disease Control and Prevention/American Academy of Periodontology). Performance varied substantially by task complexity and input richness: models predicting severe disease or high‐burden groups using dental/periodontal parameters [75, 76, 79, 80, 90, 91, 92] sometimes achieved a high AUROC (0.72 [80]–0.98 [75]), whereas predictions based primarily on surveys and biomarkers [77, 78, 81, 82, 83, 84, 85, 86, 87, 88, 94] showed more modest discrimination and higher variability (accuracy 0.72 [87]–0.91 [81], AUROC 0.60 [77]–0.98 [78]) depending on the endpoint. Sample size ranged from 110 [79] to 43 331 [92], with several studies performing external testing which did not show any major performance drops [75, 83, 85, 87] (Table S1). Although most studies implemented classical machine learning models, some recent studies employed deep learning models as well [81, 89, 91, 92, 93]. Explainability methods such as SHAP (SHapley Additive exPlanations) [78, 88, 90] and LIME (Local Interpretable Model‐agnostic Explanations) [91] were incorporated in some pipelines to rank influential predictors.

#### 3.2.3 Disease Progression and Treatment Outcome Prediction

Thirteen studies [29, 95, 96, 97, 98, 99, 100, 101, 102, 103, 104, 105, 106] addressed prognosis, progression, and treatment‐related outcomes on the basis of periodontal measures, clinical parameters, and biomarkers. Progression of periodontitis and prediction of post‐treatment pocket depth trajectories/closure were estimated using classical machine learning models and (less commonly) deep learning models in the short term (< 2 years) [29, 95, 98, 101, 103, 104], middle term (2–5 years) [96, 99, 102], and long term (> 5 years, max. 10 years) [97, 100]. Sample size also varied widely, from 75 patients [101] to 42 394 [105]. Reported discrimination was generally moderate to good (AUROC 0.58–0.89 [29, 96, 99, 100, 101, 102, 103, 104], accuracy 0.63–0.94 [29, 98, 100, 101, 102, 103, 104, 105]). Some models resulted in high sensitivity (0.75–0.89) at the cost of specificity (0.41–0.50) [95, 97], whereas others performed with low sensitivity (0.40–0.64) and high specificity (0.81–0.93) [97, 100, 101, 104], depending on the task, data characteristics, and model used. External testing, when reported, commonly showed reduced performance [29, 103], although external results were slightly better (AUROC = 0.89) compared with internal results (AUROC = 0.83) in one study [100] (Table S1).

#### 3.2.4 Oral‐Systemic and Environmental Associations

Six studies [107, 108, 109, 110, 111, 112] used AI to model links between periodontal measures and systemic or environmental variables, including hypertension and systemic inflammation (C‐reactive protein; CRP) [107, 111], cognitive impairment risk [108], heavy metal exposure [109, 110], and dietary vitamin mixtures [112]. Predictive performance ranged from modest (e.g., hypertension prediction probability 55%—sensitivity 28%, specificity 78% [107]) to stronger discrimination in exposure‐related models (e.g., heavy metal–based alveolar bone loss prediction with the best AUROC = 0.88 [109]) and biomarker‐based periodontitis prediction (e.g., AUROC = 0.82 on the development data and AUROC = 0.72 when externally tested on NHANES 2001–2004 [111]). The study investigating cognitive impairment reported perfect results; however, its relevance is limited by the sample size of 30 participants [108]. Other studies used larger datasets, ranging from 1057 to 8614 subjects [107, 109, 110, 111, 112] (Table S1).

#### 3.2.5 Biomarker Identification and Validation

Twelve studies [113, 114, 115, 116, 117, 118, 119, 120, 121, 122, 123, 124] evaluated the utility of AI for identifying and validating biomarkers for periodontal diagnosis/stratification, including gingival crevicular fluid proteomics [113, 116], peripheral blood immunologic profiles [114, 119], salivary cytokines [115, 122], metabolomics [117], proteomics [118] and inflammatory cell counts [124], microbiome sequencing [120, 121], and multi‐omics (methylation–expression integration) [123]. Training/development dataset sizes were comparably smaller than in previous sections; only half of the studies had > 100 [114, 116, 117, 120, 121, 124] samples. Reported diagnostic performance was often high in internally validated settings, whereas multi‐batch microbiome analyses reported strong but slightly reduced performance after batch‐effect adjustments [121] (Table S1). Deep learning was successfully used for the binary classification of periodontitis using gene sequences from subgingival plaque (sensitivity 0.86, accuracy 0.90) [120], as well as for automated counting of salivary oral polymorphonuclear neutrophils (sensitivity 0.98, specificity 0.97, accuracy 0.97) [124].

#### 3.2.6 Clinical Documentation (Free‐Text) Analysis

Two studies [125, 126] applied NLP to clinical documentation in electronic dental records, focusing on extracting diagnoses and tracking longitudinal change from free‐text notes. Both reported high performance against manually annotated reference standards, including near‐perfect performance metrics. Cross‐site generalization was explicitly evaluated in the study by Chuang et al., with weighted F1 scores remaining high across key entities (e.g., periodontal status, stage, and grade), though performance was lower for some attributes [126]. Additionally, a large language model was used for synthetic note generation [126]. Some of the studies listed in previous sections also used electronic dental records [92, 105], but only structured data were extracted.

## 4 Discussion

The number of publications using AI in dentistry has been steadily increasing [30, 32]. This review aimed at AI applications in periodontology and was divided into two parts. The first focused on the diagnosis of periodontitis from image data using deep learning, similar to previous reviews [21, 22, 23, 24, 25, 26, 27, 28], but with stricter eligibility criteria—most notably the requirement that the reference standard was established by periodontologists, trained dentists, or oral/maxillofacial radiologists. This approach aimed to include only studies of high quality, which likely contributed to the generally low risk of bias observed across QUADAS‐2 domains (Figure 2). However, applicability concerns were common, particularly in patient selection, because most studies leveraged datasets from a single institution, limiting model generalizability [127]. In the second part, we scoped other AI applications in periodontology, with the objective of providing a comprehensive overview of the topic. Because synthesis was complicated by substantial heterogeneity in tasks, inputs, endpoints, and reporting, we opted for a structured narrative synthesis rather than a meta‐analysis, emphasizing what is currently supported by evidence, what remains uncertain, and which methodological limitations hinder clinical translation.

In terms of image analysis, most studies focused on radiographic bone loss on panoramic radiographs and reported high performance, especially for binary discrimination of healthy cases versus periodontitis. Nevertheless, the clinical utility of a binary classification is questionable, as bone loss is a continuous spectrum, and different stages of periodontitis correspond to distinct therapeutic strategies and levels of clinical urgency. It is also noteworthy that there was heterogeneity in selecting the coronal reference point for bone loss measurement. Most studies [35, 36, 39, 40, 42, 46, 48, 52, 55, 56, 57] used CEJ as the landmark, as it is easier to standardize, whereas other studies [41, 44, 45, 49, 61] adopted a 2‐mm offset from the CEJ, which aligns more closely with the physiological crestal bone level. Furthermore, it is unclear how studies handled cases in which CEJ was obscured by crown margins or large restorations [13]. These differences/unclarities in the coronal reference point can shift the measured radiographic bone loss and influence outcomes, particularly in staging, where bone loss percentage is one of the main determinants. For instance, the offset of 2 mm decreases the measured bone loss, which may result in a lower stage than if CEJ is used as the reference point. This limits comparability across studies and underscores the importance of reporting annotator training/experience, inter‐rater agreement, and explicit labeling protocols.

The assessment of bone loss severity and periodontitis stage was slightly less accurate than binary classification on both panoramic and intraoral radiographs. This was expected, as binary tasks ignore clinical nuances between stages and are more resilient to noisy labels [128], whereas staging requires precise quantification and often suffers from class imbalance. Although there are techniques to address class imbalance, such as over−/under‐sampling (e.g., Synthetic Minority Over‐sampling Technique (SMOTE)) or class weighing, these were not consistently used or reported. As a result, however, accuracy can be misleading, because in imbalanced multi‐class settings a model may appear “accurate” while underperforming on minority classes [129]. For imbalanced data, recall (sensitivity), precision (positive predictive value), F1‐score, and area under the precision‐recall curve (AUPRC) are more informative for interpretability and deployment decisions. Other suitable metrics, which are less commonly used, include balanced accuracy (the arithmetic mean of sensitivity and specificity) or the Matthews Correlation Coefficient (MCC), which considers all four quadrants of the confusion matrix.

Bone loss was also evaluated on CBCTs, which were excluded from several recent reviews that focused only on 2D radiographs [23, 27, 28]. The three‐dimensional nature allows for more detailed analysis of periodontal defects, such as furcation involvement. Surprisingly, the identification of furcation involvement in CBCT scans [61] was less accurate than on panoramic radiographs [44], although performance in cropped CBCTs was slightly superior [60]. However, only two CBCT studies were included [60, 61] and their designs were heterogeneous, precluding making relevant conclusions. Moreover, the utility of CBCT for routine periodontal diagnosis remains questionable given the radiation dose, cost, and limited availability. In contrast, a particularly interesting direction was the diagnosis of periodontitis from intraoral photographs. This topic was recently reviewed by Mao et al. [21], but only one study using photographs fulfilled the inclusion criteria of this review, because other studies often explored gingivitis. Tao et al. trained their model on screenshots of intraoral scans and used photographs for external testing, achieving an AUROC of 93% for distinguishing healthy gingiva, gingivitis, and periodontitis [62]. This approach underscores the potential for low‐cost, photograph‐based screening in settings with limited access to radiography or specialist care.

Dataset sizes ranged from a few hundred to several thousand images per modality. Smaller datasets are particularly prone to model overfitting, meaning the model learns the training data too well, including noise and specific details. Therefore, the model performs poorly on new, unseen data, which is why external testing is important to test the generalizability of the model. Unfortunately, only four studies [39, 51, 56, 62] tested the model on data from an external source, even though some datasets are publicly available and could be used for benchmarking. Public datasets (Kaggle [50], Tufts [42], Dentex [47]) were employed in three studies; two studies made at least a part of their data available [38, 51]. Although some dental imagery is difficult to de‐identify and privacy concerns present a legitimate barrier to data sharing [130], adherence to the FAIR principles should be prioritized [131], because the limited reuse and openness of data constrain reproducibility. Similarly, authors are encouraged to share code. Importantly, several of the QUADAS‐2 concerns in the index‐test domain align with concrete design choices that can inflate performance estimates, including small test sets and cross‐validation strategies with potential leakage [37, 42, 48, 49, 60]. These are particularly consequential in dentistry, where repeated images per patient, tooth‐level cropping, or multi‐tooth labels can inadvertently mix correlated samples across splits unless patient‐level separation is enforced.

Annotation strategies varied; some studies relied on landmark detection, whereas others involved object detection [40, 49, 51, 59] or segmentation [36, 41, 44, 46, 52, 55, 56, 61]. Moreover, periodontitis was evaluated on a site/tooth level as well as on an image/patient level. This heterogeneity, along with model architectures (ranging from classic CNNs like YOLO or U‐Net to vision transformers) and variable choice of reported metrics, hindered quantitative synthesis. Nevertheless, annotation quality was generally high, involving two or more experts in most studies. This positive outcome may be partly attributed to the fact that studies with poor reference standards were excluded in the screening process. At the same time, credibility is reduced when reference standard methods are insufficiently described [40, 42] or rely on a single annotator [42, 50, 58, 60], because label noise and systematic bias can become embedded in the reference standard. This is crucial especially for tasks like staging, where disagreements may cluster at clinically important boundaries. Some studies leveraged clinical records instead of creating annotations de novo, which is time‐consuming and often involves the need for harmonizing inconsistent annotations from different annotators [128, 132]. On the other hand, there may be misalignment between clinical findings and what is visible on the image.

Although model performance metrics were high, this may not necessarily translate into a large clinical impact. As demonstrated in other dental domains such as AI‐assisted detection of caries [133] or periapical lesions [134], high standalone accuracy or AUROC may have only a modest effect on clinical diagnoses and decision‐making and may even be counter‐productive for some clinicians [133]. Even though some studies showed that the performance of AI models was comparable to that of human experts [13, 54], observer‐performance studies are essential because they test whether AI meaningfully changes clinicians' ability to detect pathological conditions, interpret those findings, and make correct decisions. Notably, such evaluations remain scarce for research‐developed periodontal models, which are rarely implemented in routine practice; therefore, we also summarized available validation and observer‐performance evidence from commercial tools to provide the best current indication of how AI may affect diagnostic performance in real‐world settings. Diagnocat (DGNCT, Miami, FL, USA) was used in six studies. On panoramic radiographs, it exhibited good to almost perfect agreement with experts (ICC = 0.76 [135], κ = 0.79 [136], κ = 0.96 [137]). On CBCTs, one study reported that the assistance of Diagnocat significantly improved dentists' sensitivity in bone loss detection (from 0.45 to 0.72) without a major adverse effect on specificity (0.81 and 0.84 with and without AI assistance, respectively) [138]. In contrast, the outcomes of two other studies on CBCTs were less positive—Feltraco et al. reported fair agreement between Diagnocat and two radiologists (κ = 0.38 and κ = 0.31) [139], and Schulze et al. found it to be ineffective in identifying periodontal lesions [140]. Another commercial tool, Second Opinion (Pearl, West Hollywood, CA, USA), improved the accuracy of interns in bone loss detection on periapical radiographs (0.81 and 0.94 with and without AI assistance, respectively) [141], whereas Denti.AI (Dover, DE, USA) did not significantly improve dentists' performance in bone loss detection on intraoral radiographs [142].

Beyond imaging, the reviewed evidence showed that AI can support periodontal care across diagnosis/classification, screening and risk stratification, prognosis and outcome prediction, oral–systemic association modeling, biomarker discovery, and automated clinical documentation analysis (Table S1). Across these tasks, the same methodological issue emerges: high internal performance is frequently reported in single‐cohort settings, but generalizability depends on data heterogeneity and consistent reference standards, resulting in inferior performance on external data. This was most marked in the study by Bashir et al., in which AUROC decreased from 0.97–0.99 to 0.50–0.59 in external testing [67], but similar patterns would likely be observed in other studies that did not test models externally, especially those with low sample sizes (Table S1).

For screening and risk stratification models, larger datasets and standardized inputs (e.g., surveys, routinely collected systemic parameters, structured data from electronic health/dental records) appear to enable broader validation and more realistic performance estimation. However, they also raise a different translational question: whether the predicted risk can be linked to actionable clinical pathways, such as triggering a periodontal exam, prioritizing prevention counseling, or tailoring recall intervals. In this context, the use of explainability tools such as SHAP or LIME is important because they can quantify feature importance to individual predictions [78, 88, 90, 91]. Nevertheless, interpretation should remain cautious because feature importance does not establish causality and can still be cohort‐dependent. In particular, highly ranked predictors may reflect confounding, proxy variables, or local practice patterns rather than true causal drivers of disease risk, and their importance can change under dataset shift.

For prognosis and treatment outcome prediction, reported discrimination was often moderate to good (AUROC 0.58–0.89 [29, 96, 99, 100, 101, 102, 103, 104], accuracy 0.63–0.94 [29, 98, 100, 101, 102, 103, 104, 105]), which agrees with the findings of the systematic review by Chow et al. on tooth loss prediction [143]. Their review also concluded that all studies were at a high risk of bias because of inappropriate handling of missing data, inappropriate evaluation of model performance, and lack of accounting for model overfitting in evaluating model performance [143]. Although these issues persist, positive trends can be noticed in recent studies, which seem to pay more attention to validation, explainability, and calibration‐relevant metrics like Brier score [29, 103, 105]. Importantly, prognostic models often operate under clinically consequential sensitivity–specificity trade‐offs: prioritizing sensitivity may help identify “high‐risk” patients for intensified supportive periodontal care, but can increase false positives and unnecessary interventions, whereas high specificity risks missing progressing cases. These trade‐offs can be mitigated by transparent threshold selection aligned with intended use, reporting class‐wise performance beyond accuracy, and improving calibration and uncertainty quantification to support risk‐based decision thresholds. Finally, long follow‐up is desirable to capture clinically meaningful endpoints, but it increases dropout and missingness and introduces confounding from heterogeneous supportive periodontal care intensity, which can bias outcome labels and impair model validity [29].

Finally, oral‐systemic/environmental association, biomarker‐focused studies, and free‐text processing using NLP highlight both promise and typical pitfalls. Studies modeling oral‐systemic or environmental associations may help generate hypotheses and support risk stratification, but they are particularly susceptible to confounding and should be interpreted cautiously, especially when based on small cohorts or cross‐sectional data. Biomarker discovery often involves high‐dimensional signals with comparatively small clinical sample sizes (Table S1), increasing the risk of overfitting and batch effects. Therefore, transparent pre‐processing and external testing are key to establishing true diagnostic value. NLP pipelines applied to clinical documentation, although seldom used in periodontology to date [125, 126], are likely to become more frequent, as they present a pragmatic way to mine electronic health/dental records and increase clinical efficiency. Interestingly, Generative Pretrained Transformer (GPT)‐4 was also used to generate synthetic notes for model training in a cross‐site setting, which may help mitigate annotation burden if the documentation remains accurate and authentic [126].

This review has several limitations. For PICO 1, we restricted inclusion to English‐language studies, potentially creating language bias. Moreover, despite strict inclusion criteria, marked heterogeneity in dataset characteristics, task definitions, and metrics precluded quantitative analysis and limited formal comparison across modalities. For PICO 2, it can be seen as a limitation that, given the intentionally broad scope and the marked heterogeneity in clinical tasks, data sources, and reported outcomes, we did not perform a formal risk‐of‐bias assessment; therefore, the credibility and applicability of individual study findings cannot be judged with the same level of certainty as for PICO 1, and the synthesis should be interpreted primarily as a scoping overview rather than a quality‐weighted evidence appraisal. We have also excluded less practice‐oriented topics, such as the use of AI (chatbots) in education and bioinformatics studies on the basis solely of public gene‐expression databases without clinical sample collection. In addition, some potentially impactful workflow applications, such as AI‐assisted dental/periodontal charting and automated documentation, appear underrepresented in the current research literature and were therefore not covered in depth in this review. Future studies should evaluate these tools systematically, focusing on accuracy, interoperability with electronic dental records, and their effects on clinical workflow efficiency.

Looking ahead, the field would benefit from greater transparency, that is, data and code sharing, and multi‐institutional cooperation, which would enable training on diverse data as well as external testing to verify generalizability. Future studies could utilize multimodal learning, and they should follow methodological and reporting standards, such as STARD‐AI or dental‐specific guidance [129, 144]. Finally, more emphasis should be placed on clinical relevance and impact to shift periodontal AI from promising prototypes toward safe and practice‐ready tools.

## 5 Conclusions

### 5.1 Implications for Clinical Practice

AI shows promise across periodontology, with deep learning achieving strong performance for image‐based diagnosis of periodontitis, particularly for radiographic bone‐loss assessment. Emerging non‐imaging applications, such as risk stratification, prognosis and biomarker‐based modeling, and automated extraction of information from clinical records, may further support personalized care. However, limited data diversity, heterogeneous tasks and metrics, and scarce external testing raise concerns about generalizability and clinical applicability. Therefore, current evidence supports cautious, context‐specific adoption, ideally with local performance checks and clinician oversight.

### 5.2 Implications for Research

Future studies should prioritize multicenter collaboration and external testing, rigorous methodology, standardized and clinically anchored outcomes, and transparent reporting. Beyond algorithm‐centric performance, prospective studies are needed to quantify effects on diagnostic workflow, clinical decision‐making, and patient‐related endpoints.

## Author Contributions

**Antonin Tichy:** conceptualization, methodology, investigation, writing – original draft, visualization, and project administration. **Nils Werner:** conceptualization, methodology, investigation, and writing – original draft. **Helena Dujic:** conceptualization, methodology, investigation, and writing – review and editing. **Charlotte Wetzel:** investigation and writing – review and editing. **Vinay Pitchika:** validation and writing – review and editing. **Caspar Victor Bumm:** investigation and writing – review and editing. **Matthias Folwaczny:** conceptualization, methodology, supervision, and writing – review and editing. **Falk Schwendicke:** conceptualization, methodology, resources, supervision, and writing – review and editing.

## Funding

The authors have nothing to report.

## Conflicts of Interest

None. Falk Schwendicke is co‐founder of the startup dentalXrai GmbH, focusing on AI‐based diagnostics in dentistry. This review was conceived, conducted and reported independently from this activity.

## Supporting information


**Data S1:** Supplementary Table.

## Data Availability

The data that support the findings of this study are available from the corresponding author upon reasonable request.
