# Supplementary material for: Artificial Intelligence in Periodontology: A Systematic Review
Source: J Periodontal Res. 2026 Mar 30;61(6):639–61. doi: 10.1111/jre.70107 (PMC13471768; doi:10.1111/jre.70107)
Supplement: Supplementary file 1 [file JRE-61-639-s001.docx]

**Table 5**: PICO 2 – extracted information about data, tasks, models and performance metrics in the included studies (ordered by task and year of publication).

| **Study** | **Sample size** | **Clinical task** | **Data used** | **AI model / algorithm** | **Performance metrics** |
| --- | --- | --- | --- | --- | --- |
| **Diagnosis and classification** | | | | | |
| Özden et al., 2015^1^ | 150 total (100 training, 50 testing) | Classification of periodontitis (healthy, gingivitis, localized chronic, generalized chronic, localized aggressive, generalized aggressive) | Clinical parameters: GR, GI, PI, PD, CAL Demographics: age, gender, education level, smoking status; RBL(categorized by clinician: 1=none, 2=≤30% sites, 3=≥30% sites) | SVM with RBF kernel; DT; ANN | SVM: Acc=0.98; DT: Acc=0.98; ANN: Sens=0.46, Correlation coefficient=0.4601 |
| Arbabi et al., 2018^2^ | 190 patients (160 training, 30 testing) | Periodontitis diagnosis and severity classification | Clinical parameters: Age, gender, PD, CAL, PI | Feedforward NN with two training algorithms: LM and SCG | LM algorithm: MSE=0.0098, Overall R=0.9054; SCG algorithm: MSE=0.055, Overall R=0.6444 |
| Farhadian et al., 2020^3^ | 300 patients (160 gingivitis, 60 localized, 80 generalized periodontitis) | Classification of periodontitis (gingivitis, localized, generalized) | Clinical parameters: age, sex, smoking, attachment loss, PI, PD, GI, alveolar RBL score, PBI, tooth mobility, oral hygiene index | SVM (linear, polynomial, radial, sigmoid kernels) | Best (radial SVM): Overall Acc=0.89, (gingivitis= 0.96, localized= 0.64, generalized=0.92) |
| Kim et al., 2020^4^ | 692 subjects (144 healthy, 548 periodontitis); External: 45 subjects | Severity prediction and classification of chronic periodontitis (healthy, moderate, severe) | Biomarkers from mouthwash/saliva samples: Copy numbers of 9 periodontal pathogens measured by multiplex qPCR (P. gingivalis, T. forsythia, T. denticola, Prevotella intermedia, F. nucleatum, C. rectus, A. actinomycetemcomitans, P. anaerobius, E. corrodens); Clinical parameters: CAL, PD, GI, PI | Four ML algorithms: feed-forward NN, RF, SVM, regularized LR; 5-fold cross-validation | Group 1 (healthy vs. moderate and severe): Average Acc=0.93, AUROC=0.96, Sens=0.96, Spec=0.81; Group 2 (healthy vs. slight, moderate and severe): Average Acc=0.90, AUROC=0.94, Sens=0.95, Spec=0.69; Group 3 (healthy vs. slight): Average Acc=0.78, AUROC=0.82, Sens=0.71, Spec=0.84 External testing: Group 1 average Acc=0.82, Group 2 average Acc=0.71, Group 3 average Acc=0.65 |
| Bashir et al., 2022^5^ | 7,138 total (Taiwan 3,453; NHANES 3,685) | Periodontitis diagnosis/prediction | Demographics: age, sex, education; Health behaviors: smoking, alcohol; Metabolic health: BMI, waist circumference, systolic/diastolic BP, fasting plasma glucose, serum triglycerides, HDL cholesterol; Oral health: dental visit in last year, mobile teeth (self-reported), floss use | 10 algorithms: AdaBoost, ANN, DT, Gaussian Process, kNN, Linear SVM, LDA, LR, RF, Naïve Bayes | Internal validation (best performers): Taiwan-RF: AUROC=0.97-0.99, Acc=97.5-99.3%; NHANES-kNN: AUROC=1.00, Acc=1.00; NHANES-RF: AUROC=0.98, Acc=0.98. External testing (all algorithms): AUROC=0.50-0.59, Acc=0.48-0.62 (large drop across all algorithms) |
| Lakshmi et al., 2023^6^ | 1000 subjects | Classification and prediction of chronic periodontitis type (localized vs. generalized) | Clinical parameters: GI, PI, tooth mobility, furcation involvement, RBL, black triangle; Demographics: age, gender, number of teeth; Behavioral: smoking status, alcohol use, pan and betel nut consumption; Systemic: DM | NB, SVM, RF kNN, LR, DT; Feature selection: SelectKBest, Information Gain, Sequential Feature Selection; Ensemble methods: Bagging, Extremely Randomised Tree, AdaBoost, Gradient Boost, Histogram-Based GB, Voting Classifier, XG Boost | 80-20% split: NB (Acc=0.96), SVM (Acc=1.00), RF (Acc=1.00), kNN (Acc=0.99), LR (Acc=1.00), DT (Acc=0.99) |
| Teja Sri et al., 2023^7^ | 100 patients | Classification of dental diseases (gingivitis, periodontitis, caries, abscess, pulpitis, stomatitis) | Clinical parameters: GP01-GP28 (periodontal/gingival parameters at different sites based on 29 columns with numerical values 0-1) | Five supervised ML algorithms compared: Naïve Bayes, LR, SVM, ANN, DT. Primary focus on SVM classifier. Feature selection and preprocessing applied. | SVM classifier results shown in confusion matrix, performance metrics were not explicitly reported in extracted pages. |
| Alshehri et al., 2024^8^ | 300 subjects | Diagnosis and classification of periodontitis (staging and grading) | Clinical parameters: BOP, GI, PI, PD, CAL, RBL, mobility, furcation involvement, GR, missing teeth; Demographics: age, sex, marital status, education, physical activity; Systemic: DM, cardiovascular status, BMI, smoking | RF, SVM, ANN, LR, DT, kNN, NB | Stage prediction: Best-performing model - RF (Acc=1.00, AUROC=1.00); Grade prediction: Best-performing models - RF (Acc=1.00, AUROC=1.00), ANN (Acc=1.00, AUROC=1.00), and LR (Acc=1.00, AUROC=1.00) |
| Ameli et al., 2024^9^ | 309 patients (training: 277, testing: 32 new unseen patients) | Classification of periodontitis stage (I-IV) and grade (A-C) | Clinical parameters: PD, CAL, BOP, PI, bleeding count; Textual clinical notes containing: medical history, dental history, blood pressure, heart rate, smoking status, DM, tooth mobility, bone loss, calculus, stain, previous periodontal surgery, allergies | Two approaches: (1) BERT with LSTM layers; (2) RegEx (regular expression) feature extraction + MLP | Best-performing BERT Model - Test set: Staging Acc=0.66, Grading Acc=0.72. Stage-specific: Stage I=1.00, Stage II=0.25, Stage III=0.72, Stage IV=0.62. Grade-specific: Grade B=0.82, Grade C=0.59. |
| Farhadian et al., 2024^10^ | 300 patients (age 18-67 years; training 200, test 100) | Classification/prediction: Predict three ordinal stages of periodontitis severity (gingivitis, localized periodontitis, generalized periodontitis) | Clinical parameters: PD, CAL, PI, GI, PBI, simplified oral hygiene index, RBLscore: 0 for <20%, 1 for 20-50%, 2 for >50% based on radiographs), tooth mobility; Demographics: age, gender, smoking status | Two models compared: (1) Ordinal RF; (2) Continuation ratio LR | Ordinal RF: Acc=0.87; Continuation ratio logistic: Acc=0.80 Most important variables: RBL(0.2449), CAL (0.0606), PD (0.0586), Oral health (0.0496), PI (0.0396) |
| Vu et al., 2025^11^ | 10,714 subjects | Binary classification of periodontitis (presence/absence); Risk prediction and patient stratification | Clinical parameters: PD, CAL; Demographics: age (30-80 years), gender, ethnicity, education, household income, health insurance; Health factors: BMI, DM, glycohemoglobin level (HbA1c), hypertension, blood lead level, kidney function, general health condition, overnight hospitalization; Behavioral factors: smoking status, alcohol consumption, dental visit frequency, food security; Total 19 features initially, reduced to 13 after feature selection | Eleven ML classifiers tested: LR, DT, SVM, RF, Naïve Bayes, kNN, Neural Networks, GradientBoost, AdaBoost, XGBoost, CatBoost | Best-performing model CatBoost 5-fold cross-validation: AUROC=0.85, Acc=0.77, Sens=0.79, Spec=0.75 |
| Wang et al., 2025^12^ | 2,352 participants (704 without periodontitis, 1,648 with periodontitis) | Binary classification: periodontitis present/absent; Multi-class classification of periodontitis severity (healthy, mild-moderate, severe) | Serum biomarkers: TSH, FT4, TT3, TT4, FT3, and thyroid hormone ratios (FT4/FT3, FT4/TT4, FT3/TT3, TT4/FT3, TT3/FT4); Clinical parameters: age, sex, BMI, smoking status, hypertension, education, overweight status, vitamin D | Five ML algorithms: XGBoost, SVM, LR, RF, MLP; Hyperparameter optimization via 5-fold cross-validation and grid search | Binary classification (XGBoost): AUROC=0.85, Sens=0.76; Multi-class (XGBoost): AUROC=0.82 |
| **Risk stratification, screening and clinical decision support** | | | | | |
| Shariff et al., 2020^13^ | Development: 6,757; Validation: 3,447 | Identification and prediction of individuals in upper quintile (80th percentile) of periodontal attachment loss by age group; patient stratification for periodontitis severity | Clinical parameters: proportion of teeth with PD ≥4 mm at incisors and molars, proportion of teeth with visible GR ≥2 mm; Demographic variables: sex, race/ethnicity, educational level, insurance status; Medical conditions: DM, arthritis, hypertension, thyroid disorder; Behavioral: current smoking | Age-specific LR models with stepwise selection (entry P=0.05, stay P=0.05); propensity score (PS) calculation | Development cohort: AUROC=0.85-0.986, Sens=0.75-0.97, Spec=0.80-0.94, Accuracy not reported for DC; Validation cohort: AUROC=0.84-0.983, Sens=0.56-0.887, Spec=0.83-0.987, Acc=0.80-0.956 |
| Shimpi et al., 2020^14^ | 11,048 (4,766 positive cases, 6,282 controls); 10% (1,104) held for validation | Periodontitis risk classification and stratification into high/low risk tiers | Clinical parameters: PD, dental calculus presence, oral hygiene status, tooth brushing/flossing frequency, number of missing teeth; Medical parameters: random blood glucose, DM, lipid panels (HDL, LDL, triglycerides, total cholesterol), systolic and diastolic blood pressure, BMI, height, weight; Demographic: age, gender, Medicaid/Medicare status; Behavioral: tobacco use status | Five supervised ML algorithms compared: Naïve Bayes, LR,SVM, ANN, DT. Feature selection using information gain with ranker method | Best-performing model (DT): AUROC=0.90, Acc=0.90, Sens=0.87, Spec=0.94 |
| Minh et al., 2022^15^ | 4,188 subjects | Predicting periodontal conditions (gingival bleeding, periodontal pockets, CPI) from sociodemographic data | Sociodemographic features (25 variables): demographics (gender, age, region, education, living area); oral hygiene habits (brushing frequency, toothpaste, dental floss, toothpick use); dental care access; food/drink consumption (fruits, sweets, soft drinks, sugary tea/coffee, sugarcane juice); smoking habits; self-assessed oral health | LightGBM, RF, LogitBoost, LR | Best-performing model (LightGBM): Periodontal pockets AUROC=0.74, CPI AUROC=0.70, Gingival bleeding AUROC=0.60, |
| Monsarrat et al., 2022^16^ | 532 subjects (mean age 33 years, range 2-83; 45% female) | Risk prediction: Identify subjects at risk of developing periodontitis using systemic and sociodemographic factors | Sociodemographic data: age, sex, educational level; Medical data: BMI, systemic pathologies (cardiovascular, endocrine, metabolic diseases), hormonal status, long-term medications; Behavioral data: smoking habits, dietary habits (dried vegetables/fruits, sugary drinks, alcohol consumption), stress; periodontal assessment: CPITN score | Machine learning pipeline: (1) BorutaPy feature selection (selected 9/30 variables), (2) Min-max scaler, (3) MLP; (4) SHAP for model explainability; Five-fold cross-validation | AUROC: CPITN 0=0.79, CPITN 1-2=0.62, CPITN 3-4=0.87 |
| Ossowska et al., 2022^17^ | 110 subjects | Patient stratification and periodontitis grading; Risk assessment for progression | Clinical parameters: PD, CAL, BOP, approximal PI; Demographics: age, gender, smoking status; Site-specific measurements from 543 input features per patient | MLP, ANN; Logistic activation function and Softmax | Overall Acc=0.84 (training set); Sens=0.86, Spec=0.80; Grade-specific: Healthy 0.80, Grade A 1.00, Grade B 0.80, Grade C 0.80; Female Acc=0.91; Male Acc=0.75 |
| Patel et al., 2022^18^ | 18,553 subjects (80% training, 20% testing) | Multi-class risk prediction for periodontitis (Healthy, mild periodontitis, and severe periodontitis) | 74 features: Demographics (age, sex, race, insurance), medical history (cardiovascular, nephrology, neurology, hematology, endocrinology, rheumatology, pulmonary, gastroenterology, infectious diseases, cancer, osteoporosis), social/behavioral factors (tobacco, alcohol, recreational drugs), clinical parameters (number of teeth, DMFT, DMFS, BOP, PI, RBL, subgingival calculus, furcation involvement), caries risk, self-reported oral health (anxiety, chewing problems, speaking trouble, self-image, teeth grinding), dental history | XGBoost with one-vs.-all multi-class strategy; 5-fold cross-validation | Combined model: AUROC=0.72; Healthy vs. periodontitis: AUROC=0.69; Mild vs. others: AUROC=0.59; Severe vs. others: AUROC=0.71 |
| Teza et al., 2023^19^ | 2,086 participants (development 3,112, validation 771) | Risk prediction/screening for severe periodontitis | Clinical parameters: gender, education level, smoking status, DM, number of teeth, plaque score; Additional features: age, income, BMI, waist-to-hip ratio, hypertension, dyslipidemia, chronic kidney disease, alcohol consumption; Laboratory: lymphocytes, uric acid, triglycerides, cholesterol, HDL, LDL; Medication: lipid-lowering drugs | Mixed-effects LR, Recurrent NN, Mixed-effects SVM, Mixed-effects DT | Best-performing model (mixed-effects LR) validation: AUROC=0.98, Acc=0.91, Sens=0.89, Spec=0.93 |
| Xu et al., 2023^20^ | 300 type 2 DM patients (224 with periodontitis, 76 without) | Periodontitis risk prediction in T2DM patients | Clinical parameters: Age, sex, BMI, education level, monthly income; Lifestyle factors: smoking, alcohol consumption, exercise habits, brushing frequency; Laboratory values: HbA1c, total cholesterol, HDL, LD | RF, LR | RF - Validation: AUROC=0.95, Sens=0.52, Spec=0.96, Acc=0.85; LR - Validation: AUROC=0.92, Sens=0.64, Spec=0.91, Acc=0.84 |
| Beak et al., 2024^21^ | Development: 7,427 patients (KNHANES); Validation: 120 patients | Prediction of periodontitis and other periodontal parameters | Clinical parameters: age, sex, PD (for CPI scoring), Self-perceived oral health, prosthetic conditions (maxillary/mandibular), Disease duration (hypertension, dyslipidemia, DM), Lifestyle factors (drinking frequency, alcohol consumption, smoking period, smoking per day, walking days per week), Anthropometric measures (waist, BMI). | LR, SVM, RF, XGBoost, NN | Internal validation (KNHANES test set, 2,229): XGBoost: AUROC=0.82, Sens=0.35, Spec=0.92; External testing (GPMC, 120): XGBoost: AUROC=0.80 |
| Deng et al., 2024^22^ | 408 subjects (66 healthy, 342 periodontitis) | Multiclass screening and classification of periodontitis; clinical decision support for non-clinical screening | Self-reported parameters: CDC/AAP questionnaire (gum disease Q1, rating of gum/teeth health Q2, tooth cleaning Q3a, loose teeth Q4, use of floss Q7); Salivary biomarker: activated MMP-8; Biochemical marker: hemoglobin in toothpaste slurries; Self-reported gingival bleeding on brushing; Demographics: age, sex; Behavioral: smoking status | LR (binary classifiers); RF classifier (multiclass); Bootstrapping for internal validation (1000 bootstrap samples) | LR (binary classification): Periodontal health: Sens=0.93, Spec=0.81, AUROC=0.90; Gingivitis: Sens=0.86, Spec=0.83, AUROC=0.89; Periodontitis: Sens=0.80, Spec=0.93, AUROC=0.90;  RF (health/gingivitis/periodontitis): Sens=0.89 (health), 91.0% (gingivitis), 85.6% (periodontitis); Spec=0.92 (health), 92.6% (gingivitis), 97.1% (periodontitis); Acc=0.92 (health), 92.4% (gingivitis), 88.8% (periodontitis); AUROC=0.97 (micro/macro-average). |
| Enevold et al., 2024^23^ | Training: 1,476 (CAMB); External validation: 3,585 (DANHES) | Prediction of Stage III/IV periodontitis from questionnaire and demographic data | Demographic: sex, age, smoking status, education level, years of schooling; Self-reported: number of teeth remaining, dental visit frequency, periodontal treatment history, self-assessed teeth/gum condition, brushing frequency, oral hygiene aids use | XGBoost, RF, Partial Least Squares; 10-fold cross-validation | Internal validation - CAMB (best-performing model): RF AUROC=0.69, Sens=0.58, Spec=0.80;  External testing - DANHES (best-performing model): XGBoost AUROC=0.70, Sens=0.59, Spec=0.81 |
| Jin et al., 2024^24^ | 1,201 patients (70% training, 30% testing) | Risk prediction and patient stratification for periodontitis; identification of significant risk factors | Demographics: age, sex, education level, household income; Lifestyle factors: smoking status; Laboratory biomarkers: FBG, calcium, potassium, uric acid, total protein, albumin, osmosis; Medical history: hypertension, liver disease, coronary heart disease, high cholesterol | SVM with RBF kernel, RF, XGBoost, MLP | Training set:  SVM Acc=0.84, AUROC=0.65;  RF Acc=0.84, AUROC=0.70; XGBoost Acc=0.83, AUROC=0.68; Test set: Similar performance maintained. |
| Eke et al., 2025^25^ | 6205 (Development: 3,234; Validation: 2,971) | Prediction of periodontitis in dentate adults (30-79 years) using self-report measures | Self-reported oral health (4 questions: "Have gum disease", "Health of teeth and gums", "Had treatment for gum disease", "Lost bone"); Demographics (age, sex, race/ethnicity, income-to-poverty ratio, education); Smoking status; Tooth loss categories | LR with predictive risk score transformation | External testing: Sens=0.83, Spec=0.60, AUROC=0.79, Acc=0.72 |
| Kageyama et al., 2025^26^ | 2,050 adults (66 healthy, 588 periodontitis) | Screening and detection of periodontitis; multiclass classification; patient stratification | Salivary microbiota: 16S rRNA gene sequencing (V1-V2 regions, 802 bacterial taxa identified); Demographics: age, sex | LightGBM with 5-fold stratified cross-validation and SHAP analysis for feature importance | Biological definitions:  Localized: AUROC=0.81, Sens=0.76, Spec=0.75;  Intermediate: AUROC=0.85, Sens=0.83, Spec=0.76;  Generalized: AUROC=0.87, Sens=0.86, Spec=0.77.  CDC/AAP definitions:  Mild: AUROC=0.77;  Moderate: AUROC=0.78;  Severe: AUROC=0.83. |
| Liu et al., 2025^27^ | 24,871 subjects | Risk prediction and patient stratification for periodontitis severity | Clinical parameters: PD, age, gender, race, DM, tobacco usage, flossing frequency, insurance status | Deep NN with 2 hidden layers (512 nodes per layer), hyperbolic tangent activation, monotonic single-index modal regression with two-piece scale Student-t distribution | Cross-validation MSE (lowest among 9 compared models  Age 0.0233,  Gender-Male 0.1794,  Race-Black 0.9609,  DM-Yes 0.0545,  Tobacco-Yes 0.1516,  Flossing-Daily -0.0197 (-0.0337 to -0.0070),  Insured -0.0526 (-0.0814 to -0.0321) |
| Rebeiz et al., 2025^28^ | 3,347 teeth (10-50 years follow-up) | Treatment planning and clinical decision support (selecting optimal periodontal therapy: surgical vs. non-surgical); tooth loss risk prediction | Tooth factors: tooth location (FDI system), RCT status, post presence, therapy type received (NST, corrective surgery, regenerative surgery, root resection, root separation);  Clinical parameters: PD (classified as <3mm, 3-6mm, >6mm), CAL, BOP, tooth mobility, furcation involvement; RBL (<15%, 15-33%, >33%), defect morphology (vertical, horizontal, combined);  Demographics: age, sex, education; Patient factors: systemic diseases (DM, cardiovascular, bruxism), smoking status, initial tooth count, SPT intervals; | RF (selected as final model), XGBoost, LightGBM, CatBoost, Ensemble Stacking method; SMOTEEN technique for class imbalance; SHAP for feature importance | RF: Acc=0.93, AUROC=0.91/0.63. |
| Suh et al., 2025^29^ | 9632 (all ages); 5601 (>50 years) | Risk prediction and opportunistic screening for periodontitis; severity classification | Demographics: age, sex, education level, family poverty-income ratio; Lifestyle: smoking status, alcohol consumption, sleep duration, physical activity; Medical conditions: DM, hypertension, arthritis, high cholesterol, depression, overweight/obesity; Biochemical markers: tissue transglutaminase, vitamin D, glycohemoglobin, blood urea nitrogen, albumin, LDL-cholesterol, monocyte count | Deep NN, Extra-Trees Classifier, LR, kNN, LightGBM, MLP, DT, SVM (linear and nonlinear), RF Explainable AI: LIME | Deep Learning: opportunistic screening AUROC=0.86; diagnosis AUROC=0.86.  Best ML model (LGBM-Lasso): opportunistic screening AUROC=0.84; diagnosis AUROC=0.85. |
| Swinckels et al., 2025^30^ | 43,331 total (10,000 development, 33,331 validation) | Risk prediction for periodontitis; early detection support | Non-image EDR features: bleeding proportion, age, number of visits, prior preventive treatment, smoking/drug use, plaque proportion, prescriptions, health status, pain/problems, medical diseases, allergies, systolic/diastolic blood pressure, medications, weight, caries, dental treatment history (38 features total) | RF (best-performing model), LSTM, Recurrent NN, ANN, LR | Validation set: RF - AUROC=0.85, Sens=0.91, Spec=0.54;  Earlier detection (1yr): AUROC=0.84, Sens=0.91; (2yr): AUROC=0.83, Sens=0.89; (3yr): AUROC=0.82, Sens=0.89 |
| Zhao et al., 2025^31^ | 604 (90 healthy controls, 514 patients with periodontitis) | Periodontitis risk prediction/stratification | Microbiological parameters: 16S rRNA sequencing data from dental plaque (27 oral microbial taxa selected as predictive indicators after filtering from 454 taxa) | Pyramid Scene Parsing Network (deep learning model with CNN module, Pyramid Pooling module, and Output module) | MAE=0.027; Maximum absolute error=0.125; Peak-to-valley error=0.228 |
| Zhu et al., 2025^32^ | 10,661 from NHANES 2009-2014 (Training: 8,529; Validation: 1066; Test: 1066) | Prediction of severe (Stage III/IV) vs. non-severe periodontitis for home-based screening | Demographics (age, sex, race); Systemic health (DM, HbA1c, BP, lipids, BMI, cotinine); Nutrition; Self-reported oral health | LR, RF, Histogram GB Trees, SVM, 3-layer MLP | Best-performing model (LR): AUROC=0.81, Acc=0.75, Sens=0.73. |
| **Disease progression and treatment outcome prediction** | | | | | |
| Nagarajan et al., 2019^33^ | 114 patients (28 progressors, 86 non-progressors); 6-month follow-up | Risk prediction and disease progression modeling; identifying patients whose periodontitis would progress over 6 months | Serum biomarkers: MMP-1, MMP-2, MMP-9, serum amyloid P, C-reactive protein, bactericidal permeability-increasing protein, lipopolysaccharide binding protein, IgG antibodies to A. actinomycetemcomitans, P. gingivalis, C. rectus, F. nucleatum, P. intermedia, T. denticola, T. forsythia, isoprostane; Saliva biomarkers: IL-1β, IL-6, TNF-α, IFN-α, MMP-8, PGE2; Plaque microbiological parameters: total bacteria, P. gingivalis, T. denticola, T. forsythia levels | SVM, NB, LDA; Ensemble predictive modeling framework with bootstrapping, feature selection (LR), pairs of biomarkers as features, majority voting | Ensemble framework - NB: Sens=0.89, Spec=0.46; SVM: Sens=0.83, Spec=0.50; LDA: Sens=0.83, Spec=0.48. |
| Lee et al., 2023^34^ | 7,840 patients (483 healthy, 313 periodontitis) | Tooth loss prediction in periodontitis patients | Clinical parameters: Age, sex, DM, osteoporosis, smoking, medication oral hygiene behaviors, periodontal diagnosis, BOP, number of missing teeth at baseline, number of sites with PD≥5mm, number of teeth with PD≥6mm and furcation involvement, tooth mobility | Two-stage machine learning pipeline: (1) RuleFit algorithm for feature selection and rule generation using Lasso; (2) Count regression model (negative binomial with log link) using identified rules/features | Machine learning model: RMSE=2.71; Classical statistical model for comparison: RMSE=3.88 |
| Troiano et al., 2023^35^ | 515 patients, 3,157 molars | Prognostic prediction of 10-year molar loss in periodontitis patients | Clinical parameters: Age, Sex, PD, CAL, Mobility; furcation involvement, Smoking status, Abutment tooth status, Radiographic bone loss | LR, SVM, kNN, DT, RF, Artificial NN, GB, NB, Ensembled Stacking (NN + LR) | Overall molar loss (external testing, aggregated): Ensembled model: AUROC=0.73, Sens=0.40, Spec=0.87; NN: AUROC=0.72, Sens=0.67, Spec=0.63; LR: AUROC=0.65, Sens=0.75, Spec=0.44. Periodontitis-related molar loss: NN & Ensembled: AUROC=0.70, Sens=0.41, Spec=0.86 |
| Wang et al., 2023^36^ | 9,273 patients (1,557,864 sites); 80% training, 10% validation, 10% testing | Prediction of periodontal treatment effectiveness at site level (post-treatment PD prediction) | Patient level: Age, teeth at baseline, frequency of periodontal maintenance, sex, clinician type; Tooth level: Mazza bleeding index, tooth mobility, furcation involvement; Site level: PD, CAL | Sequence-to-Sequence (Seq2Seq) deep learning model with Attention mechanism; Compared against: (1) RF, (2) Seq2Seq alone, (3) Seq2Seq+Attention+self-training, (4) Seq2Seq+Attention+MixMatch; 10-fold cross-validation | Overall: Site-level PD Acc=0.92; Pocket closure: Sens=0.99, Acc=0.92; By baseline PD: PD<5mm: Acc=0.95; PD≥5mm: Acc=0.80. RF (initial):51.2-Acc=0.69 |
| Lee et al., 2024^37^ | 7,840 subjects | Risk prediction of tooth loss phenotype in periodontitis patients | Clinical parameters: BOP percentage, PD (sites with PD≥5mm, teeth with PD≥6mm), furcation involvement (class I, II/III), tooth mobility (degree 1, 2/3), number of missing teeth at baseline, number of decayed teeth; Periodontal diagnosis (1999 classification); Demographic: age, gender; Medical history: DM, osteoporosis, use of low-dose aspirin, use of statins; Behavioral: smoking, brushing frequency, flossing frequency | Two-step machine learning pipeline: RuleFit algorithm + LR | ML model: AUROC=0.71, Sens=0.67, Spec=0.63; Classical model: AUROC=0.70, Sens=0.65, Spec=0.67 |
| Santamaria et al., 2024^38^ | Development: 1,626 teeth in 69 patients; Validation: 2,792 teeth in 116 patients | Prediction of 10-year tooth loss and periodontal prognosis assignment | Clinical parameters: PD, mobility, furcation involvement, BOP, CAL, RBL periapical pathology (PA) | LR and NN with hyper-parameter tuning; Comparison with clinical Tooth Prognostic System | Development cohort - NN: AUROC=0.83, Sens=0.64, Spec=0.93, Acc=0.92; LR: AUROC=0.82, Sens=0.57, Spec=0.95, Acc=0.93. Validation cohort - NN: AUROC=0.89, Sens=0.98, Spec=0.52, Acc=0.55; LR: AUROC=0.89, Sens=0.91, Spec=0.68, Acc=0.69 |
| Castro dos Santos et al., 2025^39^ | 75 patients (60 development, 15 validation) | Treatment response prediction for periodontal therapy in patients with T2DM | Clinical parameters: PD, CAL, BOP, PI, number of teeth; HbA1c levels; Demographic: age, gender; Adjunctive therapy: omega-3 and aspirin use | kNN, DT, SVM, RF, XGB, CatboostLR | Best-performing models: RF AUROC=0.83, Acc=0.80, Sens=0.64, Spec=0.87; SVM: AUROC=0.84, Acc=0.77, Sens=0.55, Spec=0.87; LR: AUROC=0.86, Acc=0.80, Sens=0.77, Spec=0.81 |
| Chatzopoulos et al., 2025^40^ | 4,117 patients (75% training, 25% testing) | Prediction of significant periodontitis progression (ΔCAL ≥1mm over ≥24 months) | Demographic (4): Age, gender, race, ethnicity; Behavioral (1): Current smoking status; Systemic (2):DM diagnosis, high blood pressure; Periodontal (3): Baseline mean PD, baseline mean CAL, baseline BOP (# sites); 10 total features | RF Classifier with 5-fold cross-validation grid search for hyperparameter tuning; Complementary multivariable LR for interpretability | Test set (1,029): AUROC=0.82, Acc=0.82, Sens=0.79, Spec=0.83 |
| Feher et al., 2025^41^ | 492 patients (414 training, 78 testing) | Prediction of individual treatment response at 1-year post-treatment (achieving clinical endpoint) | Demographic (2): Age, sex; Clinical (6):Sites with PD <5mm, sites with PD ≥5mm, sites with CAL <5mm, sites with CAL ≥5mm, % sites with BOP, % sites with plaque; Microbiological (7): Total loads of Red complex (T. forsythia, P. gingivalis, T. denticola), Orange complex, Yellow complex, Green complex, Purple complex, Actinomyces species, Miscellaneous; Treatment-related (3):Daily amoxicillin dose, daily metronidazole dose, antibiotic duration; 18 total features | Primary:RF (200 trees, max depth=5, 10 features/split, 20 max leaf nodes, min 8 samples/leaf); Benchmarking:XGBoost, SVM, kNN; Bootstrap sampling, out-of-bag validation, 10-fold cross-validation | Internal testing (414): AUROC=0.93, Acc=0.85; External testing (78): AUROC=0.76, Acc=0.76 |
| Furquim et al., 2025^42^ | 273 participants (72 healthy, 201 periodontitis) | Prediction of periodontitis progression at 12 months | Clinical parameters: Age, sex, disease stage (Health/Gingivitis, Stage II, Stage III), number of missing teeth, BOP, plaque, number of deep pockets (PD ≥5mm); Salivary biomarkers: IFN-γ, IL-6, VEGF, IL-1β, IL-10, OPG, MMP-8, MCP-1, IL-8, MMP-9 | Three algorithms tested across 14 models: (1) LR (LR) using R glm function; (2) MLP NN with dropout regularization using Python TensorFlow and Keras; (3) Probabilistic Graphical Model using Python Scikit-Learn | Model 11 (Clinical+IL-1β+age+sex): PGM: AUROC=0.88, Sens=0.55, Spec=0.81, Acc=0.79; LR: AUROC=0.72, Sens=1.0, Spec=0, Acc=0.87; MLP: AUROC=0.58, Sens=0, Spec=1.0, Acc=0.87. |
| Patel et al., 2025^43^ | 42,394 patients (80% training, 20% testing) | Periodontitis progression prediction; Classification into PD progression, PD improvement, or no change | Clinical parameters from DEHR: PD, CAL, BOP, bone loss, furcation, mobility, plaque index, missing teeth, ASA classification, smoking/tobacco use, recreational drugs, behavioral, medical history, dental treatments, insurance status; SDoH from Census/CDC data: poverty rates, population density, social vulnerability index, household vulnerability, number of dental offices, travel time, fast-food restaurant access, incarceration rates, racial distribution, home ownership | XGBoost, RF, LR, Gaussian NB; NLP pipelines for data extraction from free-text clinical notes; SHAP | XGBoost: Acc=0.94, Sens=0.94, Spec=0.96; LR: Acc=0.78; RF: Acc=0.79; Gaussian NB: Acc=0.55 |
| Pawar et al., 2025^44^ | 200 patients (45 diabetic, 36 hypertensive, 119 without systemic disease) | Tooth loss prediction/prognosis | Clinical parameters: Age, Gender, DM, Hypertension, Tooth mobility grades, Furcation involvement | NB, AdaBoost, RF, NN, DT (Orange ML platform) | NB: AUROC=1.00, Acc=0.95, Spec=0.99; AdaBoost: AUROC=1.00, Acc=1.00, Spec=1.00; RF: AUROC=1.00, Acc=0.95, Spec=0.80; NN: AUROC=1.00, Acc=1.00, Spec=1.00; DT: AUROC=0.89, Acc=0.95, Spec=0.80 |
| Walter et al., 2025^45^ | Development: 746 patients; Validation: 94 patients | Treatment outcome prediction for Step II periodontal therapy: (1) Multiclass PD categorization, (2) Pocket depth improvement prediction, (3) Pocket closure prediction | Clinical parameters: Patient-level: age, sex, BMI, antibiotic treatment, periodontal family history, orthodontic treatment, DM, stress level, smoking habits; Tooth-level: FDI notation, tooth type, root number, mobility, restoration, pulp vitality, percussion/cold sensitivity; Site-level: tooth site, PD, recession, BOP, plaque, furcation involvement | Multiple ML algorithms: LR, RF, XGBoost, Multilayer perceptron; 80-20 train-test split; 10-fold cross-validation; Hyperparameter tuning via random search or HEBO; Models tuned on F1-score or Brier score | PD categorization (RF): Acc=0.85; Pocket improvement (LR): Acc=0.58, AUROC=0.63; Pocket closure (MLP): Acc=0.72, AUROC=0.74 |
| **Oral-systemic and environmental associations** | | | | | |
| Pietropaoli et al., 2020^46^ | 8,614 subjects | Risk prediction for high/uncontrolled blood pressure; Assessment of association between periodontal inflammation and hypertension | Clinical parameters: PD, CAL, BOP; PISA (calculated from PD + BOP); Demographics: age, gender, ethnicity; Biomarkers: CRP, WBC, ferritin, HbA1c, LDL-C; BP; BMI, smoking status | GB Model for variable importance and prediction; GAM with progressive adjustments; Logit GAM for odds ratios | GBM: Relative influence BOP=94.8, PISA=5.2; Prediction probability=55%, Sens=0.28, Spec=0.78; GAM C-statistic for severe PISA=0.676, Sens=0.65, Spec=0.61; Pearson correlation PISA-BOP: R²=0.74, p<.001 |
| Arévalo-Caro et al., 2025^47^ | 30 participants (20 controls, 10 MCI) | Prediction and classification of mild cognitive impairment risk based on periodontal parameters; Risk stratification | Clinical periodontal parameters:Gingival erythema, Pocket depth measurements, BOP, PI, CAL - interproximal measurements, Number of teeth present, Periodontitis diagnosis per CDC/AAP and AAP/EFP classifications. Additional variables:Age, sex, educational level | Partial Least Squares Discriminant (PLSD) model, NB. Feature selection via multiple linear regression analysis | NB model:Acc=1.00. PLSD model:Acc=0.97. |
| Chen et al., 2025^48^ | 1,057 patients (1,249 training, 417 testing after SMOTE) | Risk prediction for alveolar bone loss (periodontitis) based on environmental heavy metal exposure | Biomarkers: Urinary heavy metal concentrations (12 metals: Ba, Cd, Cs, Co, Hg, Mn, Mo, Pb, Sb, Sn, Tl, Tu measured by inductively coupled plasma mass spectrometry); Demographics: age, sex, race, PIR, BMI, education, marital status, hypertension, DM, alcohol, smoking, physical activity. Final model selected 7 features: sex, race, age, PIR, Cd, Co, Pb | RF, LR, SVM, kNN, DT, XGBoost | RF: AUROC=0.88, Acc=0.78, Sens=0.83; KNN: AUROC=0.84, Acc=0.76; XGBoost: AUROC=0.83, Acc=0.76; SVM: AUROC=0.67, Acc=0.61; LR: AUROC=0.67, Acc=0.60; DT: AUROC=0.69, Acc=0.69 |
| Li et al., 2025^49^ | 2559 adults (1424 periodontitis, 1135 controls) | Periodontitis risk prediction from heavy metal exposure | 12 heavy metal biomarkers (urinary/blood: Mo, Cd, Pb, Hg, Co, Sb, Ba, W, Tl, U); Demographics (age, sex, education, income, BMI, smoking) | NN, SVM, RF, XGBoost, LightGBM, DT, kNN + SHAP | Best (NN): AUROC=0.75, Acc=0.68, Sens=0.64 |
| Yan et al., 2025^50^ | Development: 667; External validation: 2,952 (NHANES 2001-2004) | (1) Prediction of systemic inflammation (CRP levels) from periodontal inflammation; (2) Prediction of periodontitis from systemic biomarkers | Clinical parameters: PISA, PD, CAL, BOP; Biomarkers: Serum CRP, HDL, LDL, TG, TC; Demographics: Age, gender, ethnicity, BMI, smoking habits | RF, SVM, GB; Hyperparameter tuning via grid search; SMOTE for class imbalance; StandardScaler for feature scaling; 100 bootstrap samples for validation | Best-performing models:  CRP prediction (PISA→CRP):  SVM: AUROC=0.71 (internal), AUROC=0.74 (external).  Periodontitis prediction (Biomarkers→Periodontitis):  SVM: AUROC=0.82 (internal), AUROC=0.72 (external) |
| Zhang et al., 2025^51^ | 7708 (4236 no/mild periodontitis, 3,472 moderate/severe periodontitis) | Risk prediction: Association of vitamin intake with periodontitis prevalence | Dietary vitamin intake data: Vitamins A, B1, B2, B6, B12, C, D, E, K (from 24-hour dietary recalls); Clinical periodontal examination: PD, CAL; Covariates: age, sex, BMI, ethnicity, education, income, smoking, alcohol, physical activity, hypertension, DM | Generalized Linear Model (GLM) with LR; Bayesian Kernel Machine Regression (BKMR) with Markov Chain Monte Carlo (MCMC, 20,000 iterations); Inverse Probability of Treatment Weighting (IPTW) | GLM: Vitamin A (OR=0.90), Vitamin E (OR=0.95); BKMR: Posterior Inclusion Probability (PIP) - Vitamin A: 1.000, Vitamin E: 0.976 |
| **Biomarker identification and validation** | | | | | |
| Baliban et al., 2013^52^ | Total 96 (45 healthy, 51 CP); Training: 55; Blind test sets: 20 + 21 | Identification of optimal GCF protein biomarker combinations for periodontitis diagnosis | GCF proteomic data: 432 human proteins + 30 bacterial proteins identified via high-performance liquid chromatography-tandem mass spectrometry (HPLC-MS/MS); Final model: 7 human + 3 bacterial proteins | Mixed-Integer Linear Programming (MILP) optimization model (CPLEX solver); PILOT_PROTEIN algorithm for protein identification | Cross-validation:Acc=0.99 (40/15, 30/25, 20/35 splits). Blind test (41):Overall Acc=0.98 (40/41 correct), Sens=0.95, Spec=1.00, |
| Papantonopoulos et al., 2014^53^ | 347 periodontitis patients (4 cohorts) | Classification of periodontitis patients into AgP or CP based on immunologic profile | Biomarkers from peripheral blood:Leukocyte counts (monocytes, lymphocytes, basophils, neutrophils, eosinophils), T-cell markers, Antibody levels, Some models included RBL measurements as supplementary features | ANN, MLP. Three models built: ANN1 (leukocytes), ANN2 (interleukins), ANN3 (antibody titers). Kernel density estimation for feature engineering, cross-entropy values for training. | ANN1 (leukocyte counts - best model):Acc=0.98, Sens=0.99, Spec=0.98. Inputs: CD4/CD8 ratio, neutrophils, monocytes, eosinophils. ANN2 (interleukins):Acc=0.96, Sens=0.92, Spec=0.97. Inputs: IL-1, IL-4, INF-γ, TNF-α. ANN3 (antibody titers):Acc=0.90, Sens=0.91, Spec=0.89. |
| Nagarajan et al., 2015^54^ | 80 total (40 gingivitis, 40 periodontitis) | Classification and patient stratification between gingivitis and periodontitis; identifying patient-specific biological variations | Salivary biomarkers: IL-1β, IL-6, MMP-8, MIP-1α | LDA, QDA, NB, SVM - both as traditional single classifiers and within novel Selective Voting Ensemble Approach (SVA-LDA, SVA-QDA, SVA-NB, SVA-SVM) | SVA-LDA: Acc=0.76, Sens=0.75, Spec=0.77; SVA-QDA: Acc=0.76, Sens=0.77, Spec=0.75; SVA-NB: Acc=0.76, Sens=0.77, Spec=0.75; SVA-SVM: Acc=0.76, Sens=0.73, Spec=0.79; Traditional LDA: Acc=0.78; Traditional QDA: Acc=0.76; Traditional NB: Acc=0.76; Traditional SVM: Acc=0.75 |
| Tomás et al., 2017^55^ | 150 (75 periodontally healthy controls; 75 chronic periodontitis patients) | Diagnosis and prediction of chronic periodontitis; patient stratification; development of diagnostic nomograms | Biomarkers: GCF cytokines (16 mediators); Clinical parameters: bacterial plaque level, BOP, PD, CAL; Demographics: age, gender, smoking status, education level, insurance status; Medical conditions: DM, arthritis, hypertension, thyroid disorder | Multivariate binary LR with stepwise selection (entry/stay p=0.05); Propensity score calculation; Youden's J statistic for optimal PS cutoffs; Bootstrap internal validation (1,000 replicates) with bias correction; ROC curve analysis; Development of diagnostic nomograms | Bias-corrected performance metrics: IL1α: AUROC=0.97, Sens=0.93, Spec=0.90, Acc=0.92; IL1β: AUROC=0.96, Sens=0.92, Spec=0.94, Acc=0.93; IL17α: AUROC=0.93, Sens=0.88, Spec=0.88, Acc=0.88; IL1α+IFNγ: AUROC=0.98, Sens=0.92, Spec=0.96, Acc=0.94; IL1β+IL10: AUROC=0.97, Sens=0.93, Spec=0.94, Acc=0.94; IL17α+IFNγ: AUROC=0.97, Sens=0.89, Spec=0.93, Acc=0.91; |
| Kim et al., 2021^56^ | Discovery: 221; Validation: 50 | Identification of salivary metabolite biomarkers for periodontitis diagnosis | Salivary metabolites (31 identified) measured by 1H-NMR spectroscopy: ethanol, taurine, isovalerate, butyrate, glucose, acetone, lysine, propionate, etc. | RF (1000 trees); also PCA, orthogonal partial least squares discriminant analysis for multivariate analysis | Individual biomarkers (Discovery/Validation): Ethanol AUROC=0.86/0.85, Taurine AUROC=0.77/0.82; Butyrate AUROC=0.77/0.72; Glucose AUROC=0.76/0.73; Isovalerate AUROC=0.70/0.76.  5-biomarker panel: Discovery /Validation AUROC=0.95/0.91 |
| Liu et al., 2022^57^ | Proteomics: 15; Transcriptomics: 557 | Disease classification and identification of signature biomarkers for periodontitis diagnosis | Proteomics: tandem mass tag proteomics from gingival tissue samples (5658 proteins, 115 DEPs); Transcriptomics: Gene expression data and GSE16134 datasets (9 hub DEP-encoding genes) | Lasso regression; ANN with 6 hidden layers | Training cohort (GSE10334): AUROC=0.97; Validation cohort (GSE16134): AUROC=0.88 |
| Song et al., 2023^58^ | 80 total (4 groups: middle-aged periodontitis, middle-aged type-2 DM-associated periodontitis, aged periodontitis, aged type-2 DM-associated periodontitis); | Correlation analysis between circulating biomarkers and periodontitis severity; prediction of CAL; patient stratification based on senescence-associated secretory phenotype (SASP) profile; investigation of relationship between serum SASP levels and DM-associated periodontitis severity | Biomarkers: Serum levels of 12 SASP factors; Clinical parameters: PD and CAL Demographics: age, sex, FBG; Gingival tissue: immunofluorescence staining for senescence markers (p16, p21, HMGB1) in 17 patients | ML algorithms: (1) RF for feature selection and classification modeling (identified optimal 3-gene and 6-gene models from MEblue and MEturquoise WGCNA modules); (2) SVM for optimal diagnostic classification; (3) BPNN with 15-5-1 structure for correlation modeling between SASP levels and CAL; WGCNA for identifying immune cell-associated biomarker modules | BPNN model: R=0.874, MSE=0.137; Spearman correlations for type-2 DM-associated periodontitis: IL-1β (r=0.41, p<0.01), MMP-8 (r=0.32, p=0.043), RAGE (r=0.34, p=0.034); CAL: IL-1β (r=0.40, p=0.010), IL-4 (r=-0.34, p=0.033), MMP-8 (r=0.32, p=0.042), OPG (r=-0.32, p=0.048), OPG/RANKL (r=-0.36, p=0.024), RAGE (r=0.34, p=0.033); Log-sigmoid activation function used in BPNN |
| Gu et al., 2024^59^ | 1107 plaque samples from 787 volunteers | Classification of periodontitis vs. health | 16S rRNA gene sequences from subgingival plaque (FCGR 7-mer images) | Custom CNN, AlexNet, VGG16 | Custom CNN: Acc=0.90, Sens=0.86; VGG16: Sens=0.91; AlexNet: Acc=0.90 |
| Regueira-Iglesias et al., 2024^60^ | 796 (483 healthy, 313 periodontitis) | Periodontitis diagnosis (distinguishing health from periodontitis) | Salivary microbiome: 16S rRNA gene sequencing (V3-V4 region), ASVs, bacterial taxa abundance data from saliva samples | Sparse Partial Least Squares-Discriminant Analysis (sPLS-DA) combined with Centered Log-Ratio (CLR) transformation; Multiple batch effect removal methods tested | All samples: Training - AUROC=0.94, Sens=0.91, Spec=0.87, Acc=0.89; Testing - AUROC=0.95, Sens=0.87, Spec=0.90, Acc=0.89  After BE removal (all samples): Training - AUROC=0.94, Sens=0.82, Spec=0.92, Acc=0.88;  Testing - AUROC=0.95, Sens=0.79, Spec=0.91, Acc=0.86 |
| Blanco-Pintos et al., 2025^61^ | Development: 85 patients (44 healthy, 41 periodontitis) | Diagnostic performance of salivary protein biomarkers to detect periodontitis; patient stratification by periodontal status | Clinical parameters: PD, BOP, CAL, BPL; Salivary protein biomarkers quantified by sequential window acquisition of all theoretical mass spectra: keratin type II cytoskeletal 1, protein S100-A8, β-2-microglobulin, neutrophil defensin 1, lysozyme C, ubiquitin-60S ribosomal protein L40, isoform 2 of tropomyosin α-3 chain, resistin; 377 proteins quantified total | GAM for predictive modeling; Single-protein models (unadjusted and adjusted for age/smoking); Two-protein combination models; Internal validation by bootstrapping | Single-protein unadjusted: bc-AUROC=0.85-0.914, bc-Acc=0.79-0.86, bc-sensitivity=0.62-0.87, bc-specificity=0.61-0.98; Age-adjusted: bc-AUROC=0.97-0.992, bc-Acc=0.94-0.98, bc-sensitivity=0.90-0.98, bc-specificity=0.94-0.97; Smoking-adjusted (years): bc-AUROC=0.91-0.952, bc-Acc=0.84-0.90; Smoking-adjusted (pack-year): bc-AUROC=0.90-0.959, bc-Acc=0.84-0.90; Two-protein combinations: bc-AUROC=0.96-0.997, bc-Acc=0.92-0.96, bc-sensitivity=0.88-0.96, bc-specificity=0.90-0.97% |
| Jin et al., 2025^62^ | Training: 24; Validation: 557 | Diagnosis and classification of periodontitis; identification of diagnostic biomarkers; patient stratification based on immune microenvironment | Biomarkers: DNA methylation profiles (23,528 differentially methylated sites; Gene expression data (1,641 differentially expressed genes); Immune cell profiling; 132 candidate genes showing correlated methylation-expression patterns identified through weighted gene co-expression network analysis | Machine learning algorithms: (1) RF for feature selection and classification modeling; (2) SVM for optimal diagnostic model construction | Training dataset: 3-gene model AUROC=0.83, 6-gene model AUROC=0.82; Validation dataset 1 (GSE16134, 310): 3-gene model AUROC=0.78, 6-gene model AUROC=0.86; Validation dataset 2 (GSE10334, 247): 3-gene model AUROC=0.75, 6-gene model AUROC=0.82; Overall Acc=0.92 |
| Soheili et al., 2025^63^ | Training: 4,617 oPMNs;  Testing: 51 saliva samples | Quantification of oral inflammatory load via oPMN counting in saliva for PD severity assessment (5 levels: healthy, gingivitis, mild, moderate, severe) | Biomarker: oPMNs isolated from saliva samples; brightfield microscopy images | YOLOv4 (Deep NN); also tested: LightGBM, RF, LogitBoost, LR; k-means clustering for oral inflammatory load classification | DNN (YOLOv4): Sens=0.98, Spec=0.97; Oral inflammatory load classification: Acc=0.78-0.81 |
| **Clinical documentation (free-text) analysis** | | | | | |
| Patel et al., 2023^64^ | 28,908 patients (final dataset); Observation period: up to 15 years (2005–2019) | (1) Automated extraction of PD diagnoses from free-text clinical notes; (2) Automated classification of patients into disease progression, improvement, or no change cohorts; (3) Tracking periodontitis change over time from longitudinal EDR | Clinical parameters from free-text periodontal evaluation forms: PD type (gingivitis/periodontitis), severity (mild, mild-to-moderate, moderate, moderate-to-severe, severe), location (maxilla, mandible, tooth number), onset (acute/chronic), extent (localized/generalized); Periodontal charting data | (1) PD Extractor.py: NLP algorithm using NER with Approximate String Matching (ASM) and Levenshtein Distance Function; Python libraries: Pandas, TensorFlow, PyTorch, NLTK; (2) PD Change Classifier.py: Rule-based classification algorithm using NLTK, regular expressions, Pandas | PD Extractor.py: Acc=0.98, Sens=0.98, Spec=0.97; PD Change Classifier.py: Acc=0.98, Sens=0.99, Spec=0.98 |
| Chuang et al., 2025^65^ | Development: 8,125 records; Validation: 7,000 records | NER for periodontal diagnosis extraction from clinical notes; identification of periodontal status, stage, grade, extent, and subtype | Clinical text notes from EDRs; clinical parameters documented in free-text: PD, CAL, BOP; synthetic clinical notes generated by GPT-4 for model training | GPT-4 (Generative Pretrained Transformer 4) for synthetic note generation; RoBERTa base model fine-tuned for NER task; SpaCy tokenization; 8:1:1 train/validation/test split | Site 1 - Periodontal status: Acc=0.96; Stage: Acc=0.98; Grade: Acc=0.98; Extent: Acc=0.92; Subtype: Acc=0.95. Site 2 - Periodontal status: Acc=0.95; Stage: Acc=0.97; Grade: Acc=0.98; Extent: Acc=0.84; Subtype: Acc=0.99 |

AAP – American Academy of Periodontology; Acc – accuracy; AI – artificial intelligence; ANN – artificial neural network; AUROC – area under the receiver operating characteristics curve; AUROC – area under the ROC curve; BERT – bidirectional encoder representations from transformers; BMI – body mass index; BOP – bleeding on probing; BP – blood pressure; BPNN - back-propagation neural networks; CAL – clinical attachment level; CAMB – Copenhagen Aging and Midlife Biobank; CDC – Centers for Disease Control and Prevention; CPITN – community periodontal index of treatment needs; CNN – convolutional neural network; CRP – C-reactive protein; DANHES – Danish Health Examination Survey; DL – deep learning; DM – diabetes mellitus; DMFT/S – decayed, missing, filled tooth/surface; DT – decision tree; EDR – electronic dental records; EFP – European Federation of Periodontology; FBG – fasting blood glucose; FDI – Fédération Dentaire Internationale (World Dental Federation); FT3/4 – free T3/4; GAM – generalized additive model; GB – gradient boosting; GCF – gingival crevicular fluid; GI – gingival index; GR – gingival recession; IL – interleukin; kNN – k-nearest neighbors; LDA – linear discriminant analysis; LDL/HDL – low-/high-density lipoprotein; LM – Levenberg–Marquardt; LR – logistic regression; LSTM – long short-term memory; MIP – macrophage inflammatory protein; ML – machine learning; MLP – multilayer perceptron; MMP – matrix mettaloproteinase; MSE – mean square error; NB – naive Bayes; NER – named entity recognition; NHANES – National Health and Nutrition Examination Survey; NMR – nuclear magnetic resonance; NN – neural network; NLP – natural language processing; NST – non-surgical therapy; oPMN – oral polymorphonuclear neutrophils; PCA – principal component analysis; PCR – polymerase chain reaction; PD – probing depth; PBI – papilla bleeding index; PI – plaque index; PISA - periodontal inflamed surface area; QDA – qualitative dana analysis; RAGE – receptor for advanced glycation end products; RANKL – receptor activator of nuclear factor kappa-B ligand; RBF – radial basis function; RBL – radiographic bone loss; RCT – root canal treatment; RF – random forest; RMSE – root mean square error, ROC – receiver operating characteristic; SCG – scaled conjugate gradient; Sens – sensitivity; SHAP – SHapley Additive exPlanations; SMOTEEN – Synthetic Minority Over-sampling Technique + Edited Nearest Neighbors; Spec – specificity; SPT – supportive periodontal therapy; SVM – support vector machine; TSH – thyroid-stimulating hormone; TT – total T3/4; WBC – white blood count; XGBoost – extreme gradient boosting, YOLO – You Only Look Once

**References:**

1.	Ozden FO, Ozgonenel O, Ozden B, Aydogdu A. Diagnosis of periodontal diseases using different classification algorithms: a preliminary study. *Nigerian Journal of Clinical Practice.* 2015;18(3):416-421.

2.	Arbabi S, Jahantigh FF, Moghadam SA. Presenting a Model for Periodontal Disease Diagnosis Using Two Artificial Neural Network Algorithms. *Health Scope.* 2018;7(3).

3.	Farhadian M, Shokouhi P, Torkzaban P. A decision support system based on support vector machine for diagnosis of periodontal disease. *BMC Research Notes.* 2020;13(1):337.

4.	Kim E-H, Kim S, Kim H-J, et al. Prediction of Chronic Periodontitis Severity Using Machine Learning Models Based On Salivary Bacterial Copy Number. *Frontiers in Cellular and Infection Microbiology.* 2020;10:571515.

5.	Bashir NZ, Rahman Z, Chen SLS. Systematic comparison of machine learning algorithms to develop and validate predictive models for periodontitis. *Journal of Clinical Periodontology.* 2022;49(10):958-969.

6.	Lakshmi TK, Dheeba J. Predictive Analysis of Periodontal Disease Progression Using Machine Learning: Enhancing Oral Health Assessment and Treatment Planning. *International Journal of Intelligent Systems and Applications in Engineering.* 2023;11(10):660-671.

7.	Teja Sri N, Harika K, Harshini J, Spoorthi M. Expert system application design for enhanced dental disease diagnosis. *Journal of Cardiovascular Disease Research.* 2023;14(10):741-748.

8.	Alshehri A, Dahman M, Assiri M, et al. A decision support system based on classification algorithms for the diagnosis of periodontal disease. *Saudi Journal of Oral Sciences.* 2024;11(3):181-192.

9.	Ameli N, Firoozi T, Gibson M, Lai H. Classification of periodontitis stage and grade using natural language processing techniques. *PLOS Digital Health.* 2024;3(12):e0000692.

10.	Farhadiayn M, Torkashvand Z, Shokouhi P, Torkzaban P. Developing a Prediction Model for Periodontal Disease Using Ordinal Random Forest. *Avicenna Journal of Dental Research.* 2024;16(3):177-183.

11.	Vu G, Mayya V, Mandhidi B, et al. Application of machine learning to predict periodontal disease in US adults: A cross-sectional analysis of NHANES 2009-2014. *Health Informatics Journal.* 2025;31(4):14604582251394617.

12.	Wang H, Tian S, Bai S, Yang C, Jiang Z, Li N. Associations Between Thyroid Function and Periodontitis: A Machine Learning Approach Using NHANES. *International Dental Journal.* 2025;75(5).

13.	Shariff J, Cheng B, Papapanou P. Age-Specific Predictive Models of the Upper Quintile of Periodontal Attachment Loss. *Journal of Dental Research.* 2020;99(1):44-50.

14.	Shimpi N, McRoy S, Zhao H, Wu M, Acharya A. Development of a periodontitis risk assessment model for primary care providers in an interdisciplinary setting. *Technology and Healthcare.* 2020;28(2):143-154.

15.	Minh NTH, Cao Binh T, Dinh Hai T, Thuy Duong N, Bui Q-T. Analysis of Periodontal Conditions in the Provinces of Vietnam: Results From the National Dental Survey. *Asia-Pacific Journal of Public Health.* 2022;34(1):87-95.

16.	Monsarrat P, Bernard D, Marty M, et al. Systemic Periodontal Risk Score Using an Innovative Machine Learning Strategy: An Observational Study. *Journal of Personalized Medicine.* 2022;12(2).

17.	Ossowska A, Kusiak A, Swietlik D. Evaluation of the Progression of Periodontitis with the Use of Neural Networks. *Journal of Clinical Medicine.* 2022;11(16):4667.

18.	Patel J, Su C, Tellez M, et al. Developing and testing a prediction model for periodontal disease using machine learning and big electronic dental record data. *Frontiers in artificial intelligence.* 2022;5:979525.

19.	Teza H, Pattanateepapon A, Lertpimonchai A, et al. Development of Risk Prediction Models for Severe Periodontitis in a Thai Population: Statistical and Machine Learning Approaches. *JMIR Formative Research.* 2023;7:e48351.

20.	Xu H-M, Shen X-J, Liu J. Establishment of models to predict factors influencing periodontitis in patients with type 2 diabetes mellitus. *World Journal of Diabetes.* 2023;14(12):1793-1802.

21.	Beak W, Park J, Ji S. Data-driven prediction model for periodontal disease based on correlational feature analysis and clinical validation. *Heliyon.* 2024;10(11).

22.	Deng K, Zonta F, Yang H, Pelekos G, Tonetti MS. Development of a machine learning multiclass screening tool for periodontal health status based on non-clinical parameters and salivary biomarkers. *Journal of Clinical Periodontology.* 2024;51(12):1547-1560.

23.	Enevold C, Nielsen CH, Christensen LB, et al. Suitability of machine learning models for prediction of clinically defined Stage III/IV periodontitis from questionnaires and demographic data in Danish cohorts. *Journal of Clinical Periodontology.* 2024;51(12):1561-1573.

24.	Jin D, Lan Q, Xu Z. Periodontitis risk factor analysis and machine learning prediction model construction based on multidimensional data. *Journal of Mechanics in Medicine and Biology.* 2024;24(9).

25.	Eke PI, Wei L, Thornton-Evans G, Borgnakke WS. Scoring algorithm for predicting periodontitis in dentate adults using self-report measures - National Health and Nutrition Examination Survey 2009-2012. *Periodontology 2000.* 2025.

26.	Kageyama S, Hama S, Furuta M, et al. Performance of salivary microbiota in detecting periodontitis using a machine learning approach. *Frontiers in Cellular and Infection Microbiology.* 2025;15.

27.	Liu Q, Wang S, Bai R, Bandyopadhyay D. A Robust Monotonic Single-Index Model for Skewed and Heavy-Tailed Data: A Deep Neural Network Approach Applied to Periodontal Studies. *arXiv:250502153.* 2025.

28.	Rebeiz T, Lawand G, Martin W, et al. Development of an artificial intelligence model for assisting periodontal therapy decision-making: A retrospective longitudinal cohort study. *Journal of Dentistry.* 2025;159.

29.	Suh B, Yu H, Cha J, Choi J, Kim J. Explainable Deep Learning Approaches for Risk Screening of Periodontitis. *Journal of Dental Research.* 2025;104(1):45-53.

30.	Swinckels L, de Keijzer A, Loos B, et al. A personalized periodontitis risk based on nonimage electronic dental records by machine learning. *Journal of Dentistry.* 2025;153.

31.	Zhao Z, Liu X, Li M, Liu J, Wang Z. Oral Microbe Community and Pyramid Scene Parsing Network-based Periodontitis Risk Prediction. *International Dental Journal.* 2025;75(2):700-706.

32.	Zhu Z, Wu X, Zhu L, et al. Development of a Machine Learning Tool for Home-Based Assessment of Periodontitis. *medRxiv.* 2025.

33.	Nagarajan R, Miller CS, Dawson D, Ebersole JL. Biologic modelling of periodontal disease progression. *Journal of Clinical Periodontology.* 2019;46(2):160-169.

34.	Lee C, Zhang K, Li W, et al. Identifying predictors of tooth loss using a rule-based machine learning approach: A retrospective study at university-setting clinics. *Journal of Periodontology.* 2023;94(10):1231-1242.

35.	Troiano G, Nibali L, Petsos H, et al. Development and international validation of logistic regression and machine-learning models for the prediction of 10-year molar loss. *Journal of Clinical Periodontology.* 2023;50(3):348-357.

36.	Wang R, Yang T, Jiao J, Cao Z, Meng H, Shi D. Deep learning improves prediction of periodontal therapy effectiveness in Chinese patients. *Journal of Periodontal Research.* 2023;58(3):520-528.

37.	Lee C, Zhang K, Li W, et al. Identifying predictors of the tooth loss phenotype in a large periodontitis patient cohort using a machine learning approach. *Journal of Dentistry.* 2024;144.

38.	Santamaria P, Troiano G, Serroni M, Araùjo T, Ravidà A, Nibali L. Exploring the accuracy of tooth loss prediction between a clinical periodontal prognostic system and a machine learning prognostic model. *Journal of Clinical Periodontology.* 2024;51(10):1333-1341.

39.	Castro dos Santos N, Mangussi A, Ribeiro T, et al. Factors influencing the response to periodontal therapy in patients with diabetes: post hoc analysis of a randomized clinical trial using machine learning. *Journal of Applied Oral Science.* 2025;33.

40.	Chatzopoulos G, Wolff L. A predictive model for significant periodontal disease progression: A large-scale cohort study. *Medicina Oral, Patologia Oral y Cirugia Bucal.* 2025.

41.	Feher B, de Souza Oliveira EH, Mendes Duarte P, Werdich AA, Giannobile WV, Feres M. Machine learning-assisted prediction of clinical responses to periodontal treatment. *Journal of Periodontology.* 2025.

42.	Furquim C, Caruth L, Chandrasekaran G, et al. Developing Predictive Models for Periodontitis Progression Using Artificial Intelligence: A Longitudinal Cohort Study. *Journal of Clinical Periodontology.* 2025;52(10):1478-1490.

43.	Patel J, Badi M, Katiyar R, et al. SDoH Impact on Periodontal Disease Using Machine Learning and Dental Records. *Journal of Dental Research.* 2025;104(10):1085-1094.

44.	Pawar AR, Malaiappan S, Yadalam PK, Ganesh PR. Evaluation of the predictors of tooth loss using artificial intelligence-based machine learning approach: A retrospective study. *Journal of Indian Society of Periodontology.* 2025;29(1):42-48.

45.	Walter E, Brock T, Lahoud P, et al. Predictive modeling for step II therapy response in periodontitis - model development and validation. *npj Digital Medicine.* 2025;8(1).

46.	Pietropaoli D, Del Pinto R, Ferri C, et al. Association between periodontal inflammation and hypertension using periodontal inflamed surface area and bleeding on probing. *Journal of Clinical Periodontology.* 2020;47(2):160-172.

47.	Arévalo-Caro C, López D, Milán JAS, et al. Periodontal Indices as Predictors of Cognitive Decline: Insights from the PerioMind Colombia Cohort. *Biomedicines.* 2025;13(1).

48.	Chen JY. Development of a machine learning model related to explore the association between heavy metal exposure and alveolar bone loss among US adults utilizing SHAP: a study based on NHANES 2015-2018. *BMC Public Health.* 2025;25(1).

49.	Li Y, Tao R, Feng Z, et al. Exploring the association between heavy metal exposure and periodontitis using interpretable machine learning models: NHANES 2009-2014. *Human and Ecological Risk Assessment.* 2025;31(7):1084-1099.

50.	Yan Y, Sharma P, Suvan J, D'Aiuto F. The Association of Periodontal Inflammation and Systemic Health Indicators: A Machine Learning Approach. *Journal of Clinical Periodontology.* 2025;52(10):1466-1477.

51.	Zhang Q, Que H, Xu S, et al. The Association of Intake of Vitamin Mixtures With Periodontitis: A Machine Learning Approach on NHANES. *Journal of Periodontal Research.* 2025;60(7):676-685.

52.	Baliban RC, Sakellari D, Li ZK, Guzman YA, Garcia BA, Floudas CA. Discovery of biomarker combinations that predict periodontal health or disease with high accuracy from GCF samples based on high-throughput proteomic analysis and mixed-integer linear optimization. *Journal of Clinical Periodontology.* 2013;40(2):131-139.

53.	Papantonopoulos G, Takahashi K, Bountis T, Loos B. Artificial Neural Networks for the Diagnosis of Aggressive Periodontitis Trained by Immunologic Parameters. *PLOS One.* 2014;9(3).

54.	Nagarajan R, Miller CS, Dawson D, Al-Sabbagh M, Ebersole JL. Patient-specific variations in biomarkers across gingivitis and periodontitis. *PLOS One.* 2015;10(9):e0136792.

55.	Tomás I, Arias-Bujanda N, Alonso-Sampedro M, et al. Cytokine-based Predictive Models to Estimate the Probability of Chronic Periodontitis: Development of Diagnostic Nomograms. *Scientific Reports.* 2017;7.

56.	Kim H-J, Song Y, Lee HA, Kim S, Chung J. Metabolic phenotyping of saliva to identify possible biomarkers of periodontitis using proton nuclear magnetic resonance. *Journal of Clinical Periodontology.* 2021;48(9):1240-1249.

57.	Liu W, Qiu W, Huang Z, et al. Identification of nine signature proteins involved in periodontitis by integrated analysis of TMT proteomics and transcriptomics. *Frontiers in Immunology.* 2022;13.

58.	Song X, Yue Z, Fan L, et al. Relationship between circulating senescence-associated secretory phenotype levels and severity of type 2 diabetes-associated periodontitis: A cross-sectional study. *Journal of Periodontology.* 2023;94(8):986-996.

59.	Gu J, Chen E, Zeng T, Chen T. *Classification of Periodontal and Health Oral Samples Using Deep Learning.* 2024.

60.	Regueira-Iglesias A, Suárez-Rodríguez B, Blanco-Pintos T, et al. The salivary microbiome as a diagnostic biomarker of periodontitis: a 16S multi-batch study before and after the removal of batch effects. *Frontiers in Cellular and Infection Microbiology.* 2024;14.

61.	Blanco-Pintos T, Regueira-Iglesias A, Relvas M, et al. Diagnostic Accuracy of Novel Protein Biomarkers in Saliva to Detect Periodontitis Using Untargeted 'SWATH' Mass Spectrometry. *Journal of Clinical Periodontology.* 2025;52(2):199-214.

62.	Jin L, Yuan Z, Liu Y. Multi-omics integration to identify immune-associated biomarkers and potential therapeutics in periodontitis. *Frontiers in Medicine.* 2025;12:1640961.

63.	Soheili F, Masoudifar N, Ebrahimi S, et al. DePerio: Deep Learning-Based Oral Inflammatory Load Quantification for Periodontal Applications. *Advanced Intelligent Systems.* 2025.

64.	Patel J, Kumar K, Zai A, Shin D, Willis L, Thyvalikakath T. Developing Automated Computer Algorithms to Track Periodontal Disease Change from Longitudinal Electronic Dental Records. *Diagnostics.* 2023;13(6).

65.	Chuang YS, Lee CT, Lin GH, et al. Cross-institutional dental electronic health record entity extraction via generative artificial intelligence and synthetic notes. *JAMIA OPEN.* 2025;8(3).
